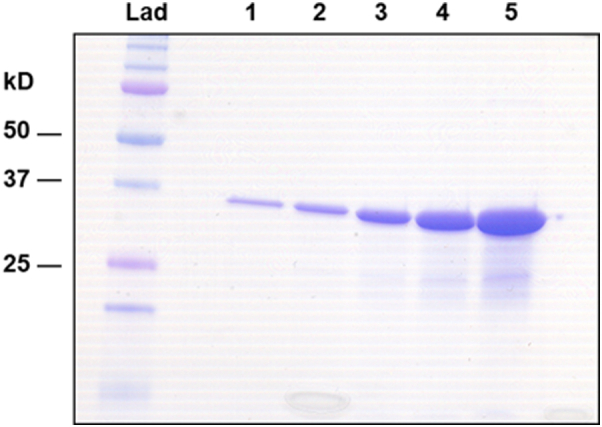# Functional genomics and structural insights into maize aldo-keto reductase-4 family: Stress metabolism and substrate specificity in embryos

**DOI:** 10.1016/j.jbc.2025.110404

**Published:** 2025-06-20

**Authors:** Sylvia Morais de Sousa, Priscila Oliveira de Giuseppe, Mario Tyago Murakami, Jiahn-Chou Guan, Jonathan W. Saunders, Eduardo Kiyota, Marcelo Leite Santos, Eric A. Schmelz, Jose Andres Yunes, Karen E. Koch

**Affiliations:** 1 Embrapa Maize and Sorghum, Sete Lagoas, MG, Brazil; 2 Brazilian Biorenewables National Laboratory (LNBR), Brazilian Center for Research in Energy and Materials (CNPEM), Campinas, SP, Brazil; 3 Department of Horticultural Sciences, University of Florida, Gainesville, Florida, USA; 4 State University of Campinas, Campinas, SP, Brazil; 5 Section of Cell and Developmental Biology, University of California at San Diego, La Jolla, California, USA; 6 Molecular Biology Laboratory, Boldrini Children's Center, Campinas, SP, Brazil

**Keywords:** crystallography, detoxification, enzyme kinetics, functional genomics, fungi, gene expression, protein structure, *Zea mays*

## Abstract

Aldo-keto reductases (AKRs) are ubiquitous in nature and are able to reduce a wide range of substrates, from simple sugars to potentially toxic aldehydes. In plants, AKRs are involved in key metabolic processes including reactive aldehyde detoxification. This study aimed to (i) delineate a maize gene family encoding aldo keto reductase-4s (AKR4s) (ii) help bridge sequence-to-function gaps among them, and (iii) focus on a family member implicated in embryo specific stress metabolism. We employed a genome-wide analysis approach to identify maize genes encoding AKR4s, defining and annotating a 15-member gene family that clustered into three subgroups. Expression profiling, validated through wet lab experiments, revealed distinct functional roles: (i) AKR4C Zm-1 functions in aldehyde detoxification during stress, (ii) AKR4C Zm-2 includes stress-responsive AKRs with diverse substrate affinities, and (iii) AKR4A/B Zm-3 contributes to specialized metabolites like phytosiderophores for iron transport. To investigate the impact of sequence variation on function, we characterized ZmAKR4C13, a representative of AKR4C Zm-1. Its mRNA and protein were predominantly localized in embryos, suggesting a specialized role. Recombinant ZmAKR4C13 efficiently reduced methylglyoxal and small aldehydes but showed poor activity toward aldoses larger than four carbons. Crystallographic analysis identified a size constraint at the active site, attributed to the bulkier LEU residue at position 294. Collectively, our results emphasize how subtle modifications in active-site architecture influence AKR substrate specificity. They also demonstrate a potential role of maize ZmAKR4C13 in detoxifying methylglyoxal and other small metabolites that could contribute to stress signaling in embryos.

Aldo-keto reductase (AKR) superfamily is widely distributed in nature and plays crucial roles in the metabolism of steroids, sugars, and other carbonyl compounds. AKRs are involved in various metabolic processes, including pentose and glucuronate interconversions, as well as the metabolism of fructose, mannose, galactose, glycerolipids, and pyruvate. They are also frequently implicated in the metabolism of both exogenous and endogenous toxicants, especially those induced by stress (1). The AKR superfamily consists of generally monomeric proteins, typically ranging from 30 to 40 kDa, that are NAD(P) (H)-dependent and share a common (α/β)_8_-or TIM-barrel (triose phosphate isomerase). Substrate specificity is primarily determined by flexible loops (A, B, and C) on the surface of core α/β–barrel. Due to the plasticity of this region, these enzymes are usually capable of accepting more than one substrate (2). Using pyridine nucleotides as cofactors, most AKRs catalyze simple oxidation–reduction reactions. The broad substrate specificity of many AKRs has made it challenging to pinpoint their precise functions. AKRs are expressed across all plant and animal species, from microorganisms to humans, with multiple forms present in each. Although AKRs have been studied relatively little in plants, the majority of plant AKRs belong to the AKR4 family, with a notable predominance in the AKR4C subfamily (subfamily delineation occurs at the 60% identity level). It has been suggested that AKR4C1–AKR4C4 help protect plant cells from desiccation by producing osmolytes that can maintain cellular integrity (3, 4, 5, 6).

Work here was motivated by the importance and diversity of AKRs, and especially those of the AKR4 subgroup. Although genes for these enzymes mediate many essential biological roles, difficulties in predicting enzyme function from gene sequence have challenged the AKR field for over 2 decades (2). Progress has been aided by programs that incorporate predictions of 3-dimensional conformations (E.G. AlphaFold by (7), but additional work is needed. A key factor has been that active sites of AKR enzymes are notoriously sensitive to subtle changes in architecture of the three flexible loops that determine substrate preference (2, 8, 9, 10). Considerable attention has been directed to the human and mammalian AKRs due to their roles in diabetes, development, and detoxification of stress metabolites (11, 12, 13, 14, 15). However, although plant AKRs also mediate critical functions, far less is known about their very different phylogeny, structure, or often elusive roles (16, 17, 18). Only three major clades of AKRs are present in plants, the AKR2’s, AKR4’s, and AKR6’s. However, the diversity of plant AKRs within these groups is highlighted by the kingdom-wide presence of at least 14 different subgroups that change in identity and abundance for angiosperms, monocots, and even in the distinctive profile of grain and grass species (19).

The plant-specific AKR4 clade has attracted the greatest research interest, yet even in this group, the gap between sequence and substrate specificity continues to leave many enzymes with largely uncertain functions (16, 17, 18). Clear delineations of this structure–function relationship have been few and far between but have been instrumental for anchoring genomic and proteomic data to activities and/or metabolic processes (20, 21, 22). Characterization of several AKR4s in dicots have revealed specific family members involved in widely diverse functions that include legume nodulation (23, 24), biosynthesis of isoflavone phenylpropanoids (with roles in nodule production) (25, 26, 27, 28), D-galacturonate reductase in strawberry (associated with ascorbate biosynthesis) (16, 29), and alteration of flavor constituents in tomato (levels of phenylacetaldehyde and 2-phenylethanol along with fruit size and sugar content (30). Roles also include alkaloid formation in *Papaver somniferum* (poppy) and *Erythroxylum coca* (coca tree) (31, 32, 33).

Considerably less is known about AKR4s in monocots, including globally important grain crops, where AKR4 phylogenies and enzyme functions can differ markedly (8, 18). A distinctive, grass-specific role, for example, has been documented for AKR4s in production of the phytosiderophores essential for iron uptake by these species (34, 35). Other AKRs examined thus far in the Poaceae have functions implicated in stress tolerance, including the abscisic acid (ABA)-inducible AKR4s in barley and *Bromus* (3, 4) and some of the AKR4s in maize and rice that can metabolize aldehydes (8, 9, 36). The emerging potential for mitigating stress metabolism is exemplified in *Echinochloa* (a rice-mimic weed) where a new pattern of expression for an AKR4 conferred a capacity for glyphosate detoxification and thus herbicide resistance (37, 38).

A key aspect of an AKR's functional role lies in its capacity to detoxify methylglyoxal (MG) and/or contribute to nitric oxide (NO) homeostasis, as both are widely recognized stress metabolites and signaling molecules across species, from humans to plants (11, 39, 40, 41). Plant AKRs have been shown to influence endogenous MG levels (8, 42) and metabolize nitric oxide derivatives (41), with broad implications for both toxicity mitigation and signal modulation. MG is initially produced as a byproduct of glycolysis or photosynthesis, forming nonenzymatically when glyceraldehyde-3-phosphate or dihydroxyacetone phosphate accumulate in excess (11, 43, 44). Its toxicity and signaling effects arise from its role as a glycating agent, modifying proteins, RNA, and DNA at specific sequence sites (11, 40). These effects can be mitigated through MG metabolism, primarily *via* glyoxalase systems I, II, and III (43, 45). However, evidence supports an alternative, backup detoxification mechanism mediated by AKRs (13, 14, 39).

In addition, AKRs are emerging as a novel class of enzymes contributing to nitric oxide homeostasis in both plants and humans (41, 46). In Arabidopsis, two AKRs are significantly upregulated in mutants with disrupted primary nitric oxide regulation, and these enzymes can reduce both S-nitrosoglutathione and S-nitroso-coenzyme A, further highlighting their potential role in nitric oxide metabolism (41, 47). The first aim here was to elucidate the phylogeny of a maize AKR4 gene family and identify subgroup associations with putative functions. To gain insight into possible roles and impacts, we profiled expression of each AKR4 at the mRNA and protein levels, and then selected six for additional depth and validation. Finally, to further delineate enzyme structure–function relationships among the AKR4s (especially those orthologous to the AKR4C Zm-1 subgroup) we focused on an ZmAKR4C13 that we found expressed during both seed maturation and in response to infection by *Aspergillus flavus*. Biochemical and structural studies of this ZmAKR4C13 showed that it could detoxify MG and other small stress-induced metabolites. Moreover, its substrate specificity was limited to small aldehydes rather than C5 or C6 aldoses, a preference based on steric hindrances imposed by a bulky leucine residue shaping the substrate-binding site. The maize ZmAKR4C13 can thus detoxify small-aldehyde stress metabolites and is expressed at sites and times in embryos conducive to a role in stress tolerance and possible signaling.

## Results

### Classification of maize AKR4s

For the first portion of this work, we delineated a 15-member gene family of aldo-keto reductase-4s (*Akr4s*) in maize by screening successive releases of the genome. AKR4C7 sequence was used as a reference to identify new AKR4 members in the maize genome. Ambiguous annotations tended to resolve with updates, but in some instances required complementary DNA (cDNA) sequences. To validate the presence of potential active sites in each gene, we aligned selected sequences with the well-studied human AKR1B1 or HsAKR1B1 (48) and aligned all 15 sequences with one another (Fig. S1). This comparison revealed that the catalytic residues Asp^44^, Tyr^49^, Lys^78^, and His^111^ (HsAKR1B1 numbering) are conserved throughout the maize AKR4 family and indicated that the genes identified were potentially active enzymes.

**Figure 1:**
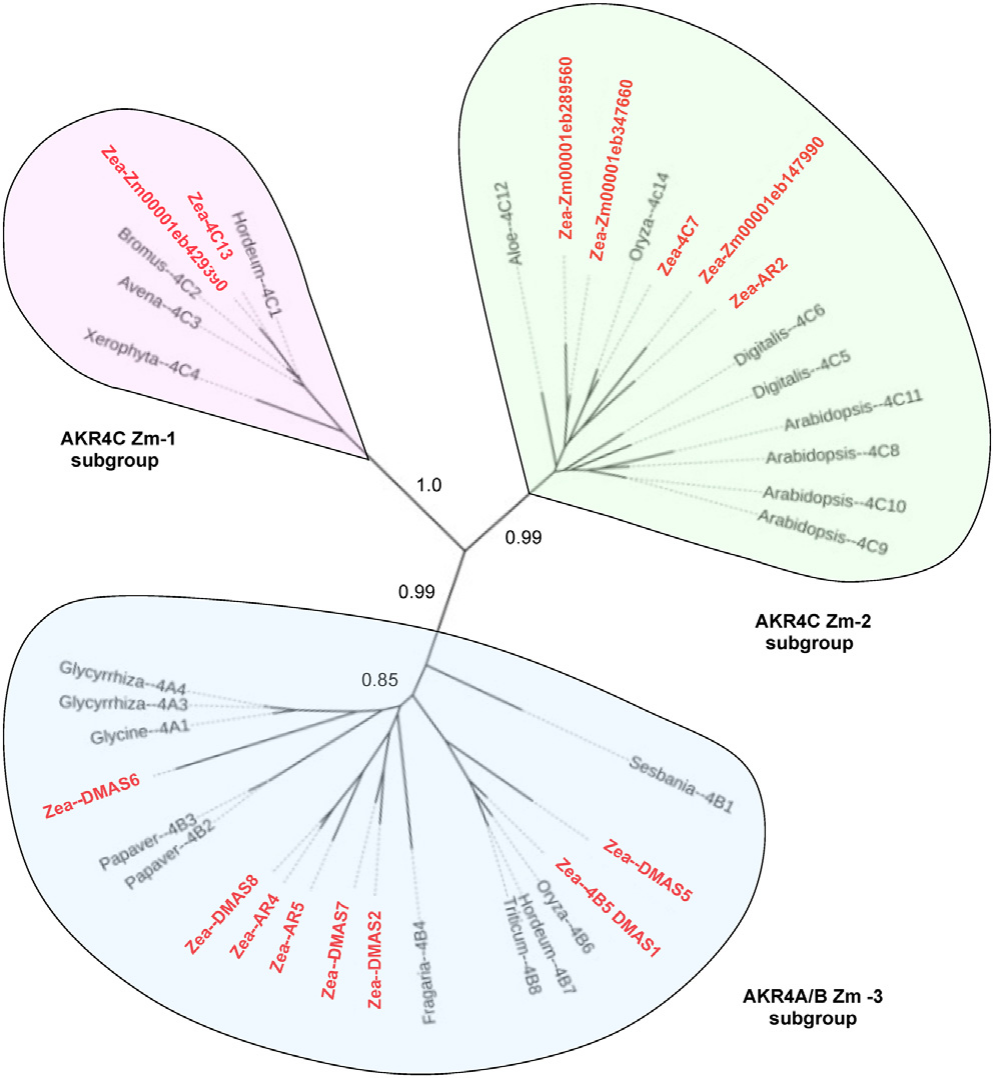
**Phylogenetic analysis of maize AKR4s with functionally-defined orthologs from other plant species.** Maize AKRs are shown in *bold red* and group into three subfamilies. The AKR4C Zm-1 Group (*pink*) are aldose reductases and include the maize ZmAKR4C13. The AKR4C Zm-2 group (*green*) includes keto reductases like the maize AKR4C7. The AKR4A/B Zm-3 (*blue*) includes the maize DMAS (AKR4B6) and AKRs for biosynthesis of complex secondary products. Sequences were aligned using ClustalX version 1.83 (76) and MUSCLE 3.7 (93). Phylogenetic relationships were determined by PhyML v. 2 at LIRMM (http://phylogeny.lirmm.fr) (78) using maximum-likelihood analysis and an approximate likelihood-ratio test for statistical evaluation of branch support values. The phylogenic tree was visualized using iTOL v. 6.5.1 (Itol.embl.de) (79). For species of origin, functional analyses, gene identifiers, and accession numbers, see Table 1. AKR, aldo-keto reductase; DMAS, deoxymugineic acid synthases.

To gain clearer insight into phylogenetic relationships among the maize AKR4s, we constructed a tree by aligning the maize sequences with others from species in which a confirmed AKR4 enzyme activity or biological function was determined for a given plant gene (Fig. 1). This approach helped anchor sequences to demonstrate functions and overcome difficulties with graminaceous phylogenies that lack some dicot clades of AKR4s (19). The maize AKR4s are shown in red and are distributed among all three of the major phylogenetic subgroups identified here for this plant-specific clade. A comparison to the kingdom-wide phylogeny of (19) showed that maize (like other grasses) is missing two clades of AKR4s ascribed to abiotic stress responses by dicots. Also, a small clade of AKR2-type genes is excluded here due to less-similar sequences, though these genes can also be classified as AKR4s depending on how group boundaries are defined (19).

Work here revealed a previously inaccessible maize clade, an AKR4C Zm-1 subgroup that had been obscured in prior genomic analyses. This subgroup is conserved among grasses and beyond, being represented in maize by the *ZmAkr4C13* and a closely related paralog. An in-depth analysis of the gene model for *ZmAkr4C13* indicated that a nearby gene, resulting in the inadvertent fusion of the two sequences, had confounded its initial annotation. The resulting merger obscured the identity of *ZmAkr4C13* as a member of the AKR4 gene clade. Additional curation at MaizeGDB.org by E. Cannon concurred with our appraisal and separated the previous gene identifier (Zm00001d038449) into two new ones: Zm00001eb290330 for the *ZmAkr4C13* (used throughout the current work) and Zm00001eb290320 for the adjacent gene (a putative member of the nexin gene superfamily). At the same time, a previously unrecognized paralog of *ZmAkr4C13* was identified and designated Zm00001eb429390. The two lie on chromosomes 6 and 10, respectively, and are currently named for their cDNAs (“*cl159_1a*” and “*cl159_b*”). A previous cDNA (GU384676) was also renamed after classification as an AKR4C. The AKR4C Zm-1 subgroup is of special interest because it is the only clade in grass and grain species (AKR4C-IVD) representing the stress-related functions that are spread among three other nongrass AKR4C clades for most angiosperms (-IVC, -IVD, and -IVE) (19). Thus far, investigations of gene function in the AKR4C subgroup 1 (IVD) have been limited to commelinid monocots including barley, oats, brome grass, and an African resurrection plant (*Xerophyta*) (Fig. 1, Table 1 and references therein).

**Table 1:** Maize AKR4 genes, proteins, and functions relative to those with demonstrated activities in other species

Zm-subgroup (kingdom-wide)	Protein name known (putative)	Species	Function and/or expression (see Figs. 2 and 3)	Analysis source	Gene identifier	Accession no.
AKR4C Zm-1 (A KR4-IVD)	4C13	*Zea mays*	Can detox methylglyoxal, little sugar metabolism.	This paper	Zm00001eb290330	
	(role unknown)	*Zea mays*	RNA only, traces in seedlings if drought	This paper, MaizeGDB.org	Zw00001eb429390	
	4C1	*Hordeum vulgare*	ABA inducible, GA responsive	(3)	HORVU1Hr1G070310	P23901.1
	4C2	*Bromus inermis*	ABA inducible, freeze tolerance	(4)		AAA21751.1
	4C3	*Avena fatua*	Desiccation, seed viability, ABA and GA	(5)		AAC49138.1
	4C4	*Xerophyta viscosa*	Desiccation inducible, tolerance	(6)		AAD22264.1
AKR4C Zm-2 (AKR4-IVB)	4C7	*Zea mays*	Can reduce aldehydes, little to no sugar	(52)	Zm00001eb148000	ABF61890
	(maize 4C9)	*Zea mays*	Can reduce aldehydes, not sugars	(49)	Zm00001eb147990	
	(AR2)	*Zea mays*	Putative AR2 (See expression, Figs. 2 and 3)	This paper, MaizeGDB.org	Zm00001eb289570	
	(role unknown)	*Zea mays*	Unknown (See expression, Figs. 2 and 3)	This paper, MaizeCDB.org	Zm00001eb289560	
	(role unknown)	*Zea mays*	Unknown (See expression, Figs. 2 and 3)	This paper, MaizeGDB.org	Zm00001eb347660	
	4C5	*Digitalis purpurea*	Can reduce specific steroids	(59)		CAC32834.1
	4C6	*Digitalis purpurea*	Can reduce specific steroids	(51)		CAC32835.1
	4C8	*Arabidopsis thaliana*	Stress aldehyde detox, NO homeostasis	(1, 70)	AT2G37760	ABH07514.1
	4C9	*Arabidopsis thaliana*	Stress aldehyde detox, NO homeostasis	(1, 70)	AT2G37770	ABH07515.1
	4C10	*Arabidopsis thaliana*	Stress aldehyde detox, NO homeostasis	(1, 70)	AT2G37790	ABH07516.1
	4C11	*Arabidopsis thaliana*	Stress aldehyde detox, NO homeostasis	(1, 70)	AT3G53880	ABH07517.1
	4C12	*Aloe arborescens*	Can reduce diverse aldehydes	Morita *et al.* 2007		ABL61257.1
	4C14	*Oryza sativa*	Can metabolize aldehydes, sugars	(36)		EEC71799
AKR4A/B Zm-3 (AKR4-IVF)	4B5; DMAS1	*Zea mays*	DMAS, Phytosiderophore biosynthesis	(34)	Zm00001eb010040	
	(DMAS2)	*Zen mays*	DMAS-like (See expression Figs. 2 and 3)	This paper. MaizeCDB.org	Zm00001eb081580	
	(DMAS5)	*Zea mays*	no data	This paper, MaizeCDB.org	Zm00001eb025990	
	(DMAS6)	*Zea mays*	DMAS-lice (See expression Figs. 2 and 3)	This paper, MaizeGDB.org	Zm00001eb419890	
	(DMAS7)	*Zea mays*	DMAS-like (See expression Figs. 2 and 3)	This paper, MaizeCDB.org	Zm00001eb423450	
	(DMAS8)	*Zea mays*	DMAS-like (See expression Figs. 2 and 3)	This paper, MaizeCDB.org	Zm00001eb423470	
	(AR4)	*Zea mays*	DMAS-like (See expression Figs. 2 and 3)	This paper, MaizeCDB.org	Zm00001eb081570	
	(AR5)	*Zea mays*	DMAS-like (See expression Figs. 2 and 3)	This paper, MaizeGDB.org	Zm00001eb101150	
	4B1	*Sesbania rostrata*	Chalcone synthase like, nodulation	(23)		CAA11226.1
	4B2	*Papaver somniferum*	Codeinone reductase	(33)		AAF13739.1
	4B3	*Papaver somniferum*	Codeinone reductase	(33)		AAF13736.1
	4B4	*Fragaria ananassa*	Can reduce D-galacturonate	(29)		AAB97005.1
	4B6	*Oryza sativa*	DMAS, Phytosiderophore biosynthesis	(34)	LOC_Os03g13390	BAF03161.1
	4B7	*Hordeum vulgare*	DMAS, Phytosiderophore biosynthesis	(34)	HORVU4Hr1G064720	BAF03162.1
	4B8	*Triticum aestivum*	DMAS, Phytosiderophore biosynthesis	(34)	Traes_4AS_887399584	BAF03163.1
	4A1	*Glycine max*	Phytoalexin biosynthesis	(27)	Glyma.14G005700	P26690.1
	4A3	*Glycyrrhiza echinata*	Polyketide reductase, forms deoxychalcone	(94)		BAA12084 1
	4A4	*Glycyrrhiza glabra*	Polyketide reductase, forms deoxychalcone	(95)		BAA13113.1

See Figure 1 for analysis of phylogenetic relationships.

Species above (common names): *Zea mays* L. (maize, corn), *Hordeum vulgare* L. (barley), *Bromus inermis* Leyss. (Brome grass), *Avena fatua* L. (common wild oat), *Xerophyta viscosa* Juss. (African resurrection plant [a commelenid]), *Digitalis purpurea* L. (Digitallis), *Arabidopsis thaliana* L. Heynh. (cress), *Aloe arborescens* Mill. (Aloe), *Oryza sativa* L. (rice), *Sesbania rostrata* Bremek. & Oberm. (a legume tree), *Papaver somniferum* L. (opium poppy), *Fragaria ananassa* Duchesne (strawberry), *Triticum aestivum* L. (common wheat), *Glycine max* L. Merr. (soybean), *Glycyrrhiza echinata* L. (liquorice), *Glycyrrhiza glabra* L. (liquorice).

Other AKR4C type enzymes cluster in the AKR4C Zm-2 subgroup (AKR4C-IVB) (Fig. 1, Table 1) and are classified as aldose reductases. These include the maize AKR4C7 or ZmAKR4C7 with a confirmed structure–function relationship for its activity (9, 49, 50), along with AKR4C5 and AKR4C6 (DpAKR4C5 and DpAKR4C6, respectively) of *Digitalis purpurea* (51) and others of *Arabidopsis thaliana* (AtAKR4C8, AtAKR4C9, AtAKR4C10, and AtAKR4C11) (Fig. 1, Table 1 and references therein). This AKR4C Zm-2 subgroup (AKR4C-IVB) has proliferated abundantly in grasses and legumes relative to other plant families (19).

Identification of a third clade, AKR4 Zm-3 subgroup in maize is consistent with recent work on a similar but kingdom-wide AKR4A/B-IVF cluster (19). The maize Zm-3 subgroup includes prominent representation of deoxymugineic acid synthases (DMAS) genes for a distinctive mechanism of iron uptake and homeostasis in grain species (34). Focus on a central, canonical DMAS enzyme has confirmed the activity of a maize AKR4B5, an *Oryza sativa* AKR4B6 or OsAKR4B6, a *Hordeum vulgare* AKR4B7 or HvAKR4B7, and a *Triticum aestivum* AKR4B8 or TaAKR7B8 (Fig. 1, Table 1 and references therein). Roles of the others are less clear despite their designations as *dmas* genes (maizegdb.org and (35). These and other maize sequences in this subgroup may have functions as diverse as those of orthologous genes that include a wide range of roles. One is the chalcone polyketide reductase activity in *Glycerrhiza echinata* (GeAKR4A3 and GeAKR4A4) and *Glycine max* (GmAKR4A1) (Fig. 1, Table 1). Another is the D-galacturonate reductase from *Frageria ananassa* (FaAKR4B4) that groups closely with two yet-to-be examined maize enzymes (Fig. 1, Table 1). Still a third function is that of the codeinone reductases PsAKR4B2 and PsAKR4B3 from *P. somniferum*.

Another small clade of enzymes somewhat distantly related to the others is involved in sugar-alcohol biosynthesis (19). In other species, these genes encode enzymes for biosynthesis of sorbitol-6-P and/or mannitol-6-P (10, 21). In maize, there are two sequences annotated as sorbitol-6-P genes. Their expression is generally not abundant and tends to predominate in vegetative tissues. These have little to no sequence similarity to the very different sorbitol dehydrogenase in maize (52).

### Localization and developmental timing of expression for maize AKR4 genes

**Figure 2:**
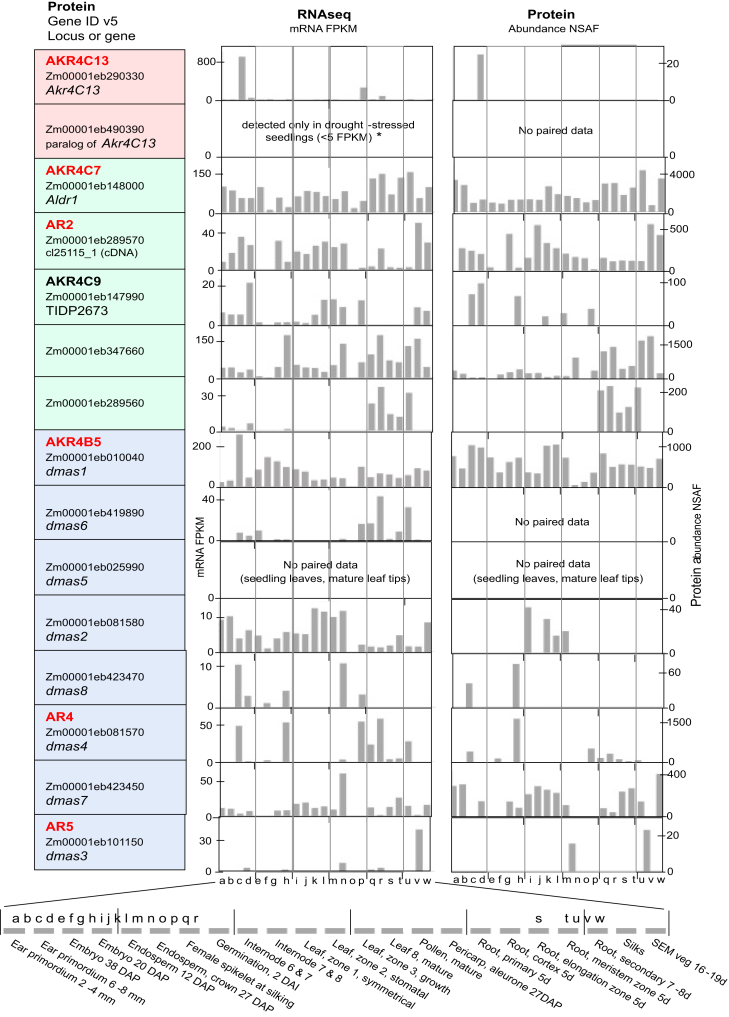
**Transcriptome and proteome comparisons for the maize AKR4 gene family.** Data were compiled from (53) accessible through qTeller (qteller.maizegdb.org) for paired samples used to quantify abundance of mRNAs (FPKM and RNAseq) and proteins (NSAF). The *left* column shows protein and enzyme names (where defined by AKR nomenclature), gene locus (as per MaizeGDB.org), and gene identifiers linked to version 5 of the maize genome. Background colors denote phylogenetic groups 1 to 3 as in Figure 1 and Table 1. Note that the *Y*-axis scale is adjusted for each family member, particularly for proteins that can vary markedly in their abundance. Tissues of origin are shown on the *X* axis, which is expanded for clarity at the base of the figure. SAM, shoot apical meristem; AKR, aldo-keto reductase.

**Figure 3:**
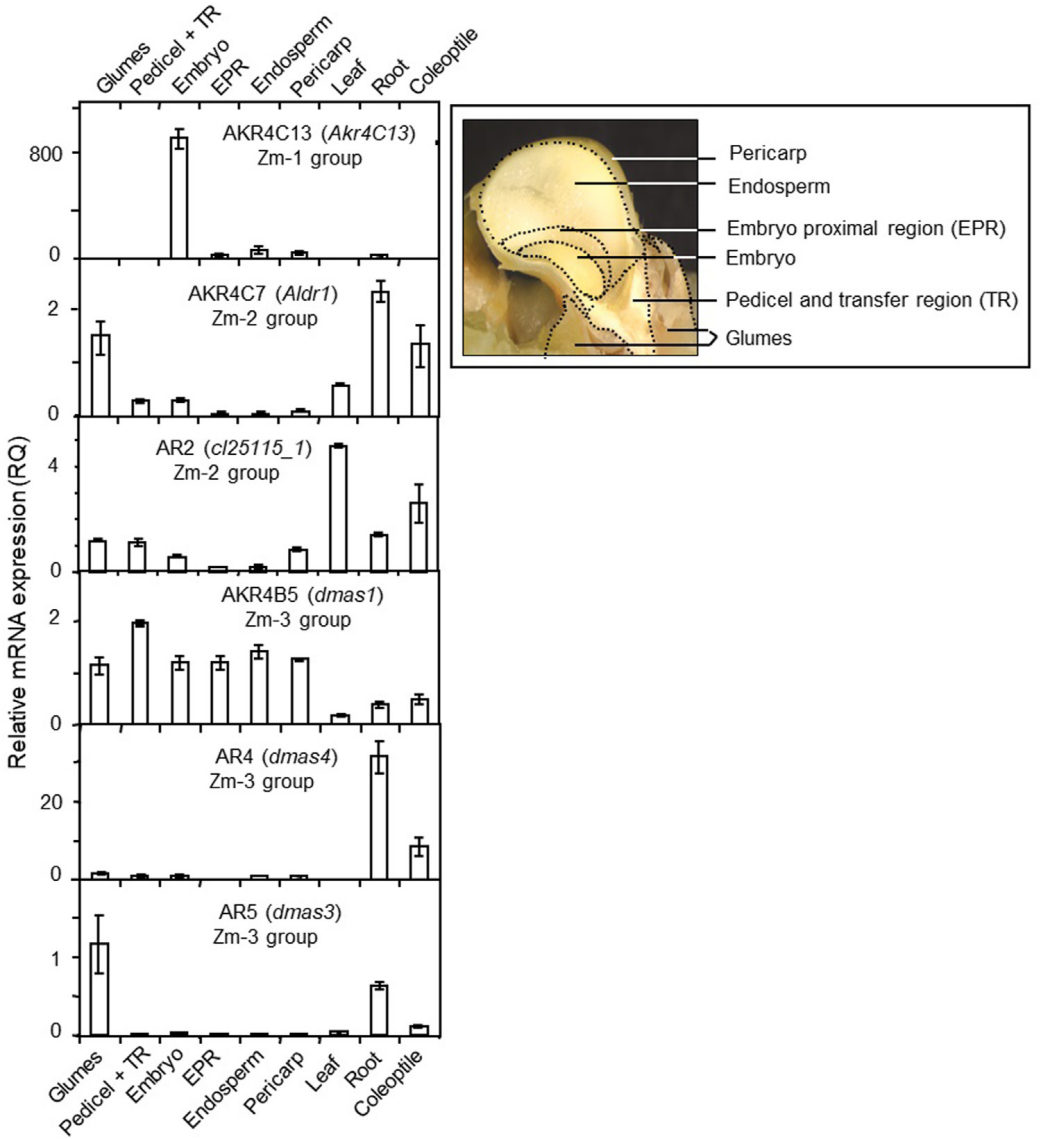
**Relative expression of six AKR genes in reproductive and vegetative tissues of WT (W22) maize as quantified by qPCR.** Kernels were sampled at 20 DAP for glumes, pedicel, and transfer region, embryo, embryo proximal region, endosperm, and pericarp (labeled in the sagittal-section visual shown). Vegetative tissues were sampled from newly germinating seedlings including leaf, root, and coleoptile. Error bars are standard error of the mean (SEM) of three independent biological replicates. AKR, aldo-keto reductase; DAP, days after pollination; qPCR, quantitative PCR.

To gain an extended, integrated appraisal of expression for maize AKR4s at both the protein and mRNA levels, we used a combination of data from public resources (Fig. 2) and results from our own gene-specific quantitative PCR (qPCR) profiles (Fig. 3). We first extracted data from (53) (accessible at qTeller.maizegeb.org) where paired samples allowed comparison of RNAseq and proteome profiling. These data were linked directly to version 5 of the maize genome, thus minimizing complexities associated with previous, less-complete genome annotations. The length and extent of conserved sequences also pose a special challenge for automated annotations of a gene family like the maize AKR4s. We therefore selected at least one member of each group for in-depth analysis using gene-specific primers for qPCR quantification of mRNA abundance (Fig. 3).

A striking embryo specificity was evident for *ZmAkr4C13* in the AKR4 Zm-1 subgroup (AKR4C-IVD) at both the transcript and protein levels (Fig. 2). An especially prominent abundance in embryos was indicated at the RNA level by the FPKM values for *ZmAkr4C13.* At the protein level, sensitivity to detection can vary by an order of magnitude or more, so comparisons there must be limited to within a given profile and not between different proteins. A strong embryo specificity was nonetheless clear when relative mRNA levels were quantified by gene-specific qPCR (Fig. 3). In other species, orthologs of this gene in the AKR4C Zm-1 subgroup are also strongly expressed in embryos, as observed for both barley (AKR4C1) (3) and oat (5).

For the other clades examined (AKR4 Zm-2 and Zm-3 subgroups), each appeared to include one or two family members that were broadly expressed while others were more specific (Fig. 2). In the AKR4C Zm-2 subgroup (AKR4C-IVB), for example, profiles for both mRNA and protein abundance extended to most above ground tissues for the AKR4C7 (*Aldr1*) and AR2 (cl25115_1). In contrast, the AKR4C9 (TIDP2673) showed an embryo-enhanced expression consistent with our earlier work on this AKR4 (de Sousa, unpublished). A root-specific maize member of the AKR4C Zm-2 subgroup was also identified (Fig. 2).

Expression of maize genes in the AKR4A/B Zm-3 subgroup (AKR4A/B-IVF) appeared to be essentially constitutive for the *AKR4B5* (*Dmas1)* that mediates biosynthesis of the iron chelators used for uptake and internal transport in grasses (34, 35). On one hand, the widespread expression observed here (Figs. 2 and 3) is consistent with broader roles proposed for iron transport and use throughout the plant (35). On the other hand, the work presented here did not include iron deficiency treatments, which in maize and rice were found to markedly upregulate expression of the *Dmas1* genes in roots (encoding AKR4C5 and AKR4B6, respectively) (34, 35). Still, data here show that the maize AKR4B5 was more strongly expressed in kernels than in root tissues. Other members of the AKR4A/B Zm-3 subgroup include DMAS-like enzymes with roles yet to be defined. Their expression patters differ in subtle and potentially important ways. Additional members of this maize subgroup that cluster phylogenetically with genes for specialized products in other species are shown here to have distinctive patterns of abundance at both the mRNA and protein levels (Figs. 2 and 3). The AR4 (DMAS-4) is expressed primarily during germination in coleoptiles and primary roots, with modest expression in kernels, whereas AR5 (DMAS-3) predominates in silks, leaves, and glumes. The latter is intriguing given that glumes of the maize ancestor, teosinte, had major roles as protective maternal structures that fully encased each kernel in a hard, nutshell like covering.

### The embryo-specific ZmAKR4C13 is upregulated during kernel development and by infection with *A. flavus*

**Figure 4:**
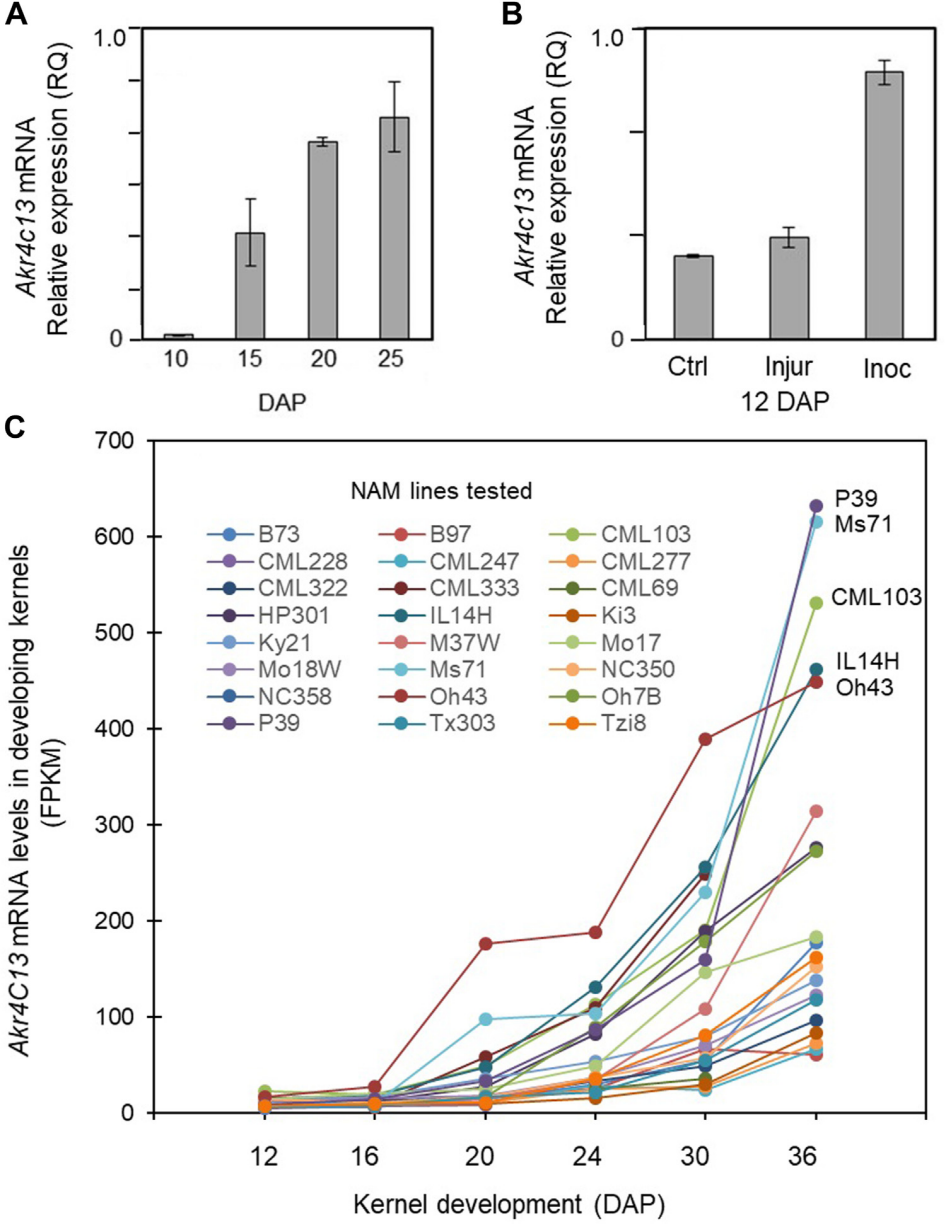
**Expression of *ZmAkr4C13* mRNA in maize kernels.***A*, relative expression during kernel development. *B*, induction of Zm*Akr4C13* by *Aspergillus flavus* in kernels at 12 DAP: Undamaged controls (Ctrl), mechanically damaged (Injur), inoculated with *A. flavus* (Inoc). *C*, genetic variation for timing and extent of *ZmAkr4C13* expression in developing kernels of 24 NAM lines. Five of these lines showed consistently higher levels of *ZmAkr4C13* mRNA throughout development and Oh43 was distinctive in its elevated expression during the peak grain-fill period (from 20 to 25 DAP). The genetic variation evident here indicates a high potential for enhancing expression of *ZmAkr4C13* through breeding. RNA-seq data were compiled from (57) after downloading from the Maize Genomics Resources (http://maize.uga.edu/gene_expr_analyses_download.shtml). For A and B: Error bars are standard error of the mean (SEM) of three independent biological replicates. AKR, aldo-keto reductase; DAP, days after pollination; NAM, nested association mapping.

The *ZmAkr4C13* mRNA profiles show that in addition to its embryo specificity (Figs. 2 and 3), its expression is detectable as early as 10 DAP (days after pollination), and rises during kernel development (Fig. 4*A*). A similar pattern was observed for this AKR ortholog from AKR4C family in barley (*HvALR1*) (54) and rice (*OsALR1*) (55), indicating a high degree of conservation. Moreover, we investigated the *ZmAkr4C13* gene expression of maize kernels in response to *A. flavus* infection, since earlier work had suggested that this fungus first colonizes the embryo and aleurone, and then spreads to the endosperm (56). Transcript levels of *ZmAkr4C13* increased significantly during *A. flavus* infection of developing kernels (12 DAP) compared to control kernels with and without mechanical damage (Fig. 4*B*). Comparisons between expression of *ZmAkr4C13* during kernel development in NAM parents were based on RNA-seq data from (57) downloaded for individual genes at http://maize.uga.edu/gene_expr_analyses_download.shtml (Fig. 4*C*). Two of the NAM founders were sweet corns (P39 and Il14h) in which levels of expression for *ZmAkr4C13* at 36 DAP were among the four highest observed for any of the 24 lines (Fig. 4*C*). Although consistent with potential for the abundant sugars in sweet corn to produce reactive MG that induce *ZmAkr4C13*, the rise in expression did not occur until well after typical sweet corn harvest (20–25 DAP). For this reason, the increases in *ZmAkr4C13* could have been due to mechanical damage that occurs during excessive shrinkage of these kernels during final maturation.

### ZmAKR4C13 prefers small-aldehyde substrates

We next characterized the recombinant ZmAKR4C13 protein to help define its *in vivo* role and capabilities. We also compared features of this protein to those of other AKRs expressed in maize embryos and especially ZmAKR4C7, the first aldose reductase identified in *Zea mays* (9). Work here shows that the ZmAKR4C13 predominates in embryos and is more strongly tissue specific. The yield of purified ZmAKR4C13 protein was three times higher than that previously obtained for ZmAKR4C7 (9). Mass spectrometry analysis determined a molecular mass of 35,659.7 Da for recombinant maize ZmAKR4C13, slightly larger than that of ZmAKR4C7, previously analyzed by (9). The activity of the recombinant maize ZmAKR4C13 enzyme remained stable, showing no significant change even after months of storage at −20 °C. Assays showed a characteristic AKR activity with reduction of a standard AKR substrate, DL-glyceraldehyde, in the presence of NADPH (Table 2). This is considered a reasonable predictor of *in vivo* activity because (1) most AKRs prefer NADPH over NADH and (2) the lack of metal or flavin cofactors leaves most AKRs relatively ineffective as alcohol dehydrogenases. In metabolically active cells, NADP^+^ is predominantly in its reduced form (58), favoring reduction over oxidation. The NADPH/NADP^+^ ratio reflects the cell’s synthetic capacity and operates independently of the NAD^+^/NADH ratio, which is primarily regulated by glycolysis and respiration. As a result, AKRs can perform their metabolic and detoxification functions without being influenced by fluctuations in cofactor levels caused by changes in metabolic rate or capacity. The consistent high levels of NADPH provide a strong driving force for AKRs to catalyze reduction across various cellular energetic states, including respiration, growth, reproduction, and starvation (12). Nonetheless, many AKR proteins can act on a broad range of substrates (1, 6, 8, 41, 51, 54, 59, 60, 60, 61). This breadth was only partially evident here for the maize ZmAKR4C13, since sugars with more than 4-C made poor substrates. Levels well above their physiological range were needed before this enzyme could use NADPH to reduce D-arabinose, D-xylose, or D-ribose (Table 2). Neither glucose nor sorbitol were effective substrates for ZmAKR4C13, in contrast to roles initially proposed for its orthologs in other grains (3, 5). However, ZmAKR4C13 has an especially high affinity (low km) for MG and p-nitrobenzaldehyde, similar to that shown previously for AKRs of *Escherichia coli* (62), humans (63) and Arabidopsis (1). Modifications of the *in vitro* assay environment using oxidized DTT did not significantly alter results (not shown).

**Table 2:** Kinetic parameters of recombinant maize aldose reductase AKR4C13

Substrate	km (mM)	Std. Error	kcat (s^−1^)	kcat/km (s^−1^ mM^−1^)
D-sorbitol^a^	ND	ND	ND	ND
D-glucose^b^	ND	ND	ND	ND
D-ribose^b^	1605.00	436.10	0.07	4.08E^−05^
D-xylose^b^	758.30	52.90	0.17	2.21E^−04^
D-arabinose^b^	286.70	57.17	0.14	4.76E^−04^
D-glyceraldehyde^b^	1.46	0.29	18.03	12.35
Methylglyoxal^b^	0.94	0.08	2.21	2.35
p-nitrobenzaldehyde^b^	0.10	0.02	0.65	6.51

ND, no detectable activity.

a Substrate was oxidized.

b Substrate was reduced.

### Overall ZmAKR4C13 structure

**Figure 5:**
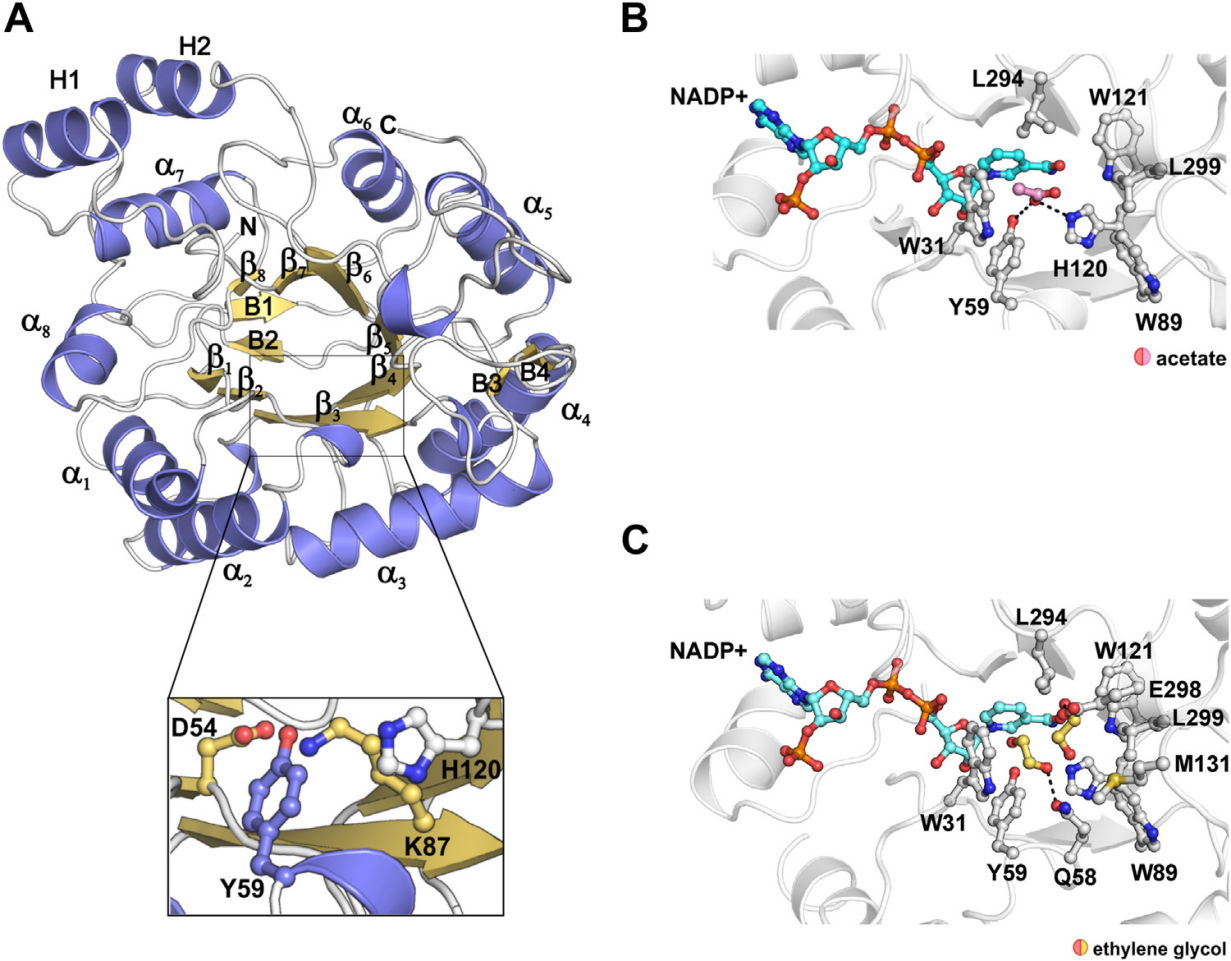
**The crystal structure of ternary complexes in ZmAKR4C13.***A*, cartoon representation of the enzyme overall structure highlighting the conserved α_8_/β_8_ folding and the catalytic tetrad (inset). *B*, the ternary complex of ZmAKR4C13 (*cartoon*) with NADP^+^ (*cyan* carbon atoms) and acetate (*pink* carbon atoms) highlighting residues (*gray* carbon atoms) from the active site that contact acetate. *C*, the complex of ZmAKR4C13 (*cartoon*) with NADP^+^ (*light blue* carbon atoms) and two molecules of ethylene glycol bound into the active site. Residues interacting with the ethylene glycol molecules are shown in the stick-and-ball model. *Dashed lines* represent hydrogen bonds. AKR, aldo-keto reductase.

To identify mechanistic features potentially affecting activity and roles of the ZmAKR4C13, we solved its crystal structure in two space groups at a resolution of 2.3 Å (*P*2_1_2_1_2_1_) and 1.45 Å (*P*2_1_), respectively (Table 3). Unfortunately, crystallization assays using the apoenzyme failed, indicating that flexible regions, stabilized only upon cofactor binding, might have prevented crystal formation. The protein folds into a (α_8_/β_8_)-barrel with two additional α-helices (H1 and H2) packed against the α7-helix from the barrel. In this respect, it resembles other AKR enzymes (20, 22, 48) (Fig. 5, *A* and *B*). The residues Asp^54^, Tyr^59^, Lys^87^, and His^120^ correspond to the typical catalytic tetrad of AKRs according to structural comparison with the well characterized AKR1C9 (64). The *P*2_1_2_1_2_1_ crystal revealed a ternary complex with NADP^+^ and acetate while the *P*2_1_ crystal displayed a NADP^+^ with partial occupancy and two molecules interpreted as ethylene glycol in the enzyme active site (Fig. 5, *C* and *D*).

**Table 3:** Data collection and refinement statistics

	AKR4C13-NADP-ACT	AKR4C13-NADP-EDO
Data collection		
Space group	*P*2_1_2_1_2_1_	*P*2_1_
Cell dimensions		
*a*, *b*, *c* (Å)	114.65, 115.47, 124.94	53.75, 115.30, 56.47
α, β, γ (°)	90	90, 104.28, 90
Resolution (Å)^a^	36.78–2.30 (2.44–2.30)	57.65–1.45 (1.53–1.45)
*R*_merge_	0.05 (0.65)	0.05 (0.64)
*I*/σ*I*	16.8 (2.2)	11.4 (1.4)
Completeness (%)	99.1 (98.2)	98.6 (98.6)
Redundancy	3.8 (3.8)	1.9 (1.9)
Refinement		
Resolution (Å)	36.78–2.30	39.73–1.45
No. reflections	69931	112014
*R*_work_/*R*_free_	0.21/0.25	0.16/0.18
No. atoms		
Protein	9766	4970
Ligand/ion	204	140
Water	126	695
*B*-factors		
Protein	62.11	17.04
Ligand/ion	59.41	12.62
Water	51.41	28.92
R.m.s. deviations		
Bond lengths (Å)	0.014	0.001
Bond angles (°)	1.490	1.854

a Values in parentheses are for highest-resolution shell.

**Figure S2:**
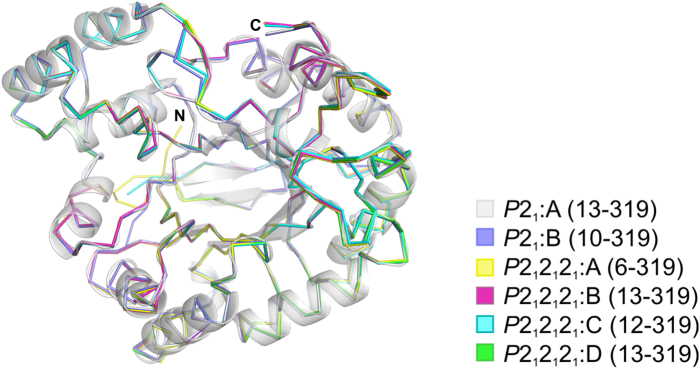


Although two and four monomers compose the asymmetric unit of the *P*2_1_ and *P*2_1_2_1_2_1_ crystals, respectively, analysis of their interfaces using the PDBePISA program (65) did not indicate formation of stable quaternary structures in solution. This result agrees with the canonical monomeric state observed for AKR enzymes (66) and could be further validated in future studies using in solution techniques such as size-exclusion chromatography or small-angle X-ray scattering. The six protein chains readily superimposed with an overall RMSD of 0.26 Å for the 307 Cα atoms aligned (Fig. S2). In all chains, some of the N-terminal residues as well as the C-terminal His-tag were not modeled due to poor electron density (Fig. S2).

### The active site

**Figure 6:**
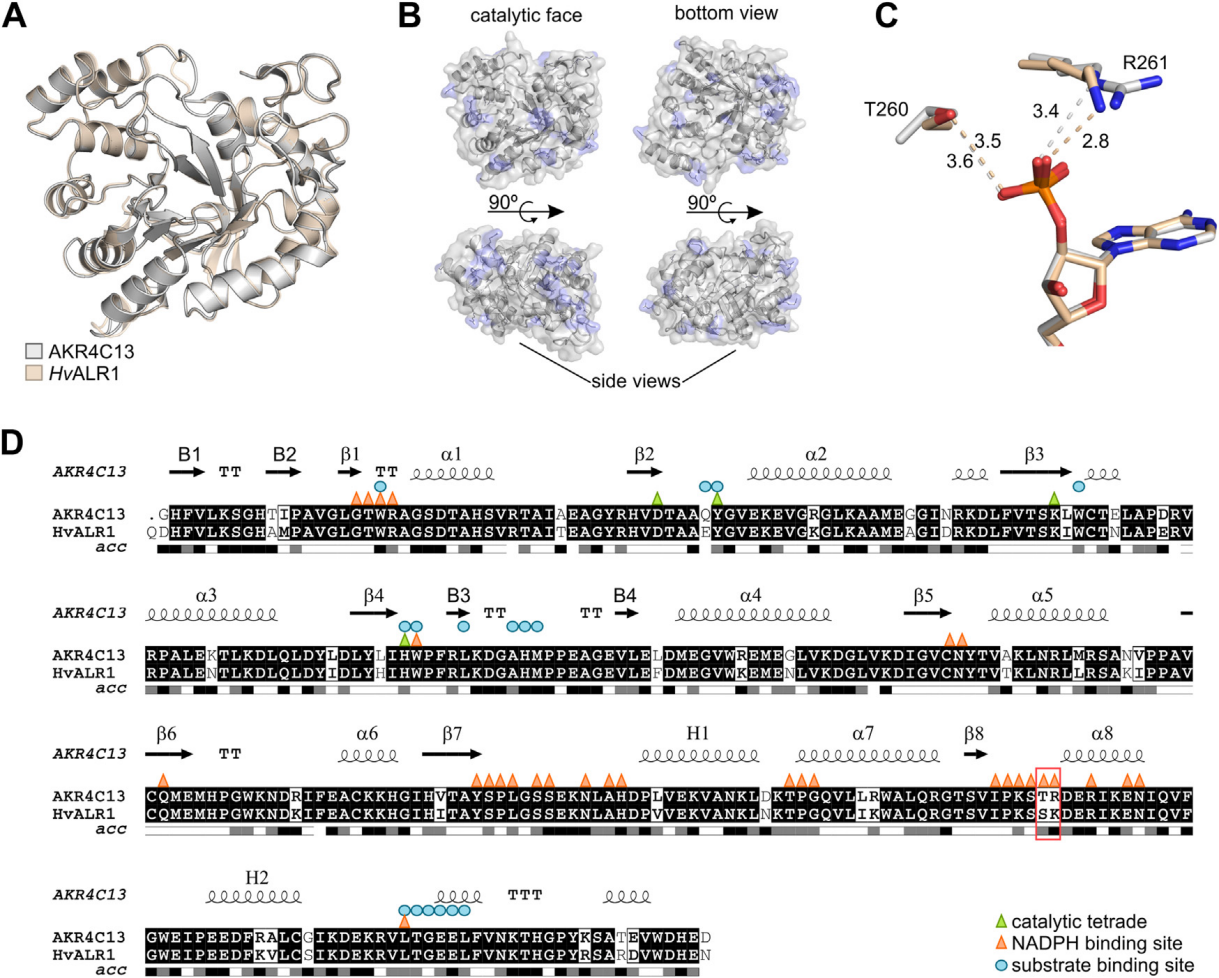
**Structural comparison of ZmAKR4C13 with a barley AR.***A*, structural superimposition of *Hv*ALR1 with ZmAKR4C13 highlighting loops A, B, and C that shape the active site. *B*, cartoon and surface representation of ZmAKR4C13 highlighting in *lilac* the residues divergent in *Hv*ALR1. *C*, conservative substitutions in the NADPH binding site preserve polar contacts (*dashed lines*, distance in Å) with the 2-phosphoryl moiety of the cofactor. *D*, sequence alignment between ZmAKR4C13 and *Hv*ALR1 highlighting the conserved sites for cofactor and substrate binding, and the T260S and R261 K substitutions (*red box*). Acc = solvent accessibility from buried (*white*) to fully exposed (*black*). The sequences were aligned using CLUSTAL Omega (93), and the image was generated using ESPript 3.0 (92). Note the location of Loops A, B and C color-coded as in *panel A*. AKR, aldo-keto reductase.

We presented the structures of two novel enzyme–substrate complexes, providing further insights into the structural determinants of substrate recognition. Structural comparisons using PDBeFold (67) identified barley AKR or HvALR1 (AKR4C1) as the closest known structural ortholog of ZmAKR4C13 (20); Protein Data Bank (PDB) ID: 2BGS). Superimposition of these enzymes yielded an RMSD of 0.49 Å across 307 aligned Cα atoms, with an 88% sequence identity (Fig. 6*A*). Most amino acid substitutions occur on the protein surface and involve conservative mutations (Fig. 6, *B* and *D*). The cofactor-binding site is identical in both enzymes, except for the T260S and R261K substitutions, which maintain polar interactions with the 2′-phosphoryl moiety of the adenosine group (Fig. 6, *C* and *D*). The substrate-binding site is also highly conserved, suggesting similar substrate specificity (Fig. 6*D*). In HvALR1, this site was characterized based on its crystal structure in complex with butanol (20).

**Figure 7:**
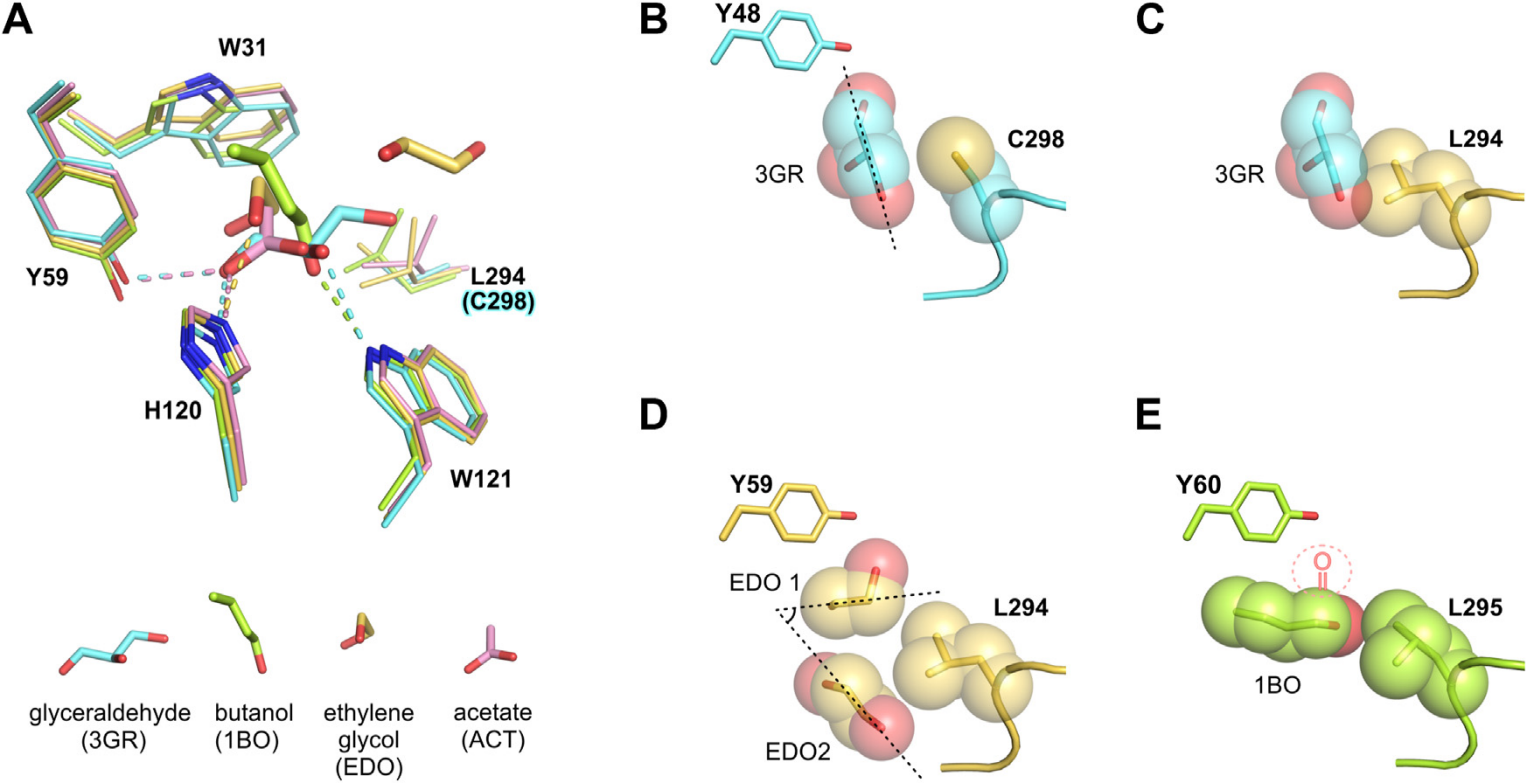
**Structural insights into the substrate specificity of ZmAKR4C13.***A*, structural comparison of AR complexes with butanol (*Hv*ALR1, *purple C atoms*, PDB ID: 2VDG); acetate (AKR4C13, *pink C atoms*, this work); ethylene glycol (AKR4C13, *yellow* C atoms, this work) and glyceraldehyde (HsAKR1B1, *cyan C atoms*, PDB ID: 3V36) highlighting the ligands bound to the substrate binding site *via* conserved hydrophobic interactions with Trp^31∗^ and hydrogen bonds with Tyr^59∗^, His^120∗^, and/or Trp^121∗^. In the analyzed plant enzymes, Leu^294∗^ also stabilizes the ligands *via* hydrophobic interactions. In the human enzyme, this residue is replaced by a cysteine (C298), which contacts the substrate glyceraldehyde. In the lower panel, ligands are represented as *sticks* in the same orientation as in the *upper* panel. *B*, Cys^298^ allows the binding of glyceraldehyde (3GR) in an orientation (*dashed lines*) not allowed by (*C*) Leu^294∗^ due to steric impediments—represented by the superimposition of the Van der Waals spheres of 3GR and Leu^294∗^ atoms. *D*, to circumvent the bulky side chain of Leu^294∗^, the ethylene glycol (EDO) molecules are accommodated at the ZmAKR4C13 active site by changing the orientation (*dashed lines*) of their carbon chains. *E*, the longer alcohol, butanol, orientates its carbon chain in a similar way to that of EDO 1, but places its OH group 2 Å further than the expected position of the substrate aldehyde group (*red dashed lines*). AKR, aldo-keto reductase; PDB, Protein Data Bank.

Two molecules of ethylene glycol were found at the substrate-binding site for ZmAKR4C13, allowing us to identify additional points of potential substrate anchoring based on hydrophobic interactions and hydrogen bonds. The first molecule occupies the same binding site as does acetate in the *P*2_1_2_1_2_1_ crystal and that of butanol in *Hv*ALR1 (Fig. 7). Comparisons of protein residues in contact with the three ligands indicate that their carbon scaffold preserves hydrophobic interactions with Trp^31∗^ (^∗^AKR4C13 numbering). Oxygen atoms of ethylene glycol and acetate form hydrogen bonds with His^120∗^ while the OH group of butanol lies in the vicinity of Trp^121∗^ and makes an H-bond with the N-H group of the tryptophan side chain. Comparing these interactions with those of the substrate glyceraldehyde in the HsAKR1B1 (PDB ID: 3V36) (68), we conclude that the acetate H-bond to His^120∗^ mimics that which orients the substrate aldehyde group, whereas the butanol H-bond to Trp^121∗^ is comparable to that made by the substrate C-2 OH group (Fig. 7*A*).

**Figure S3:**
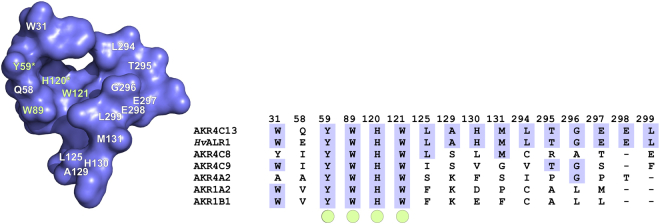


Glyceraldehyde is a common substrate for AR and binds to the most conserved region of the substrate-binding site (Fig. S3). However, this site in the more distant catalytic tetrad is highly divergent and confers a range of substrate specificities to the AR enzymes. Some of the AKRs, including ZmAKR4C13, display poor affinity for C5 monosaccharides and have lost their capacity to use glucose (C6) as a substrate (Table 2). Replacement of a cysteine by the bulkier leucine at position 294^∗^ from Loop C seems to hamper the binding of C3-OH groups that are in the same orientation observed for glyceraldehyde in HsAKR1B1 (Fig. 7, *B* and *C*). This modification correlates with an almost 80-fold higher k_m_ for the binding of longer C3-OH substrates to ZmAKR4C13 relative to the human enzyme, and it might hinder the use of longer-chain monosaccharides by ZmAKR4C13.

Supporting this hypothesis, the orientation of the second ethylene glycol molecule bound in the ZmAKR4C13 active site displays an angular shift compared to the first one (Fig. 7*D*). This second ligand forms H-bonds with Gly^296∗^ and Glu^298∗^ and hydrophobic contacts with the Met^131∗^, Leu^294∗^, and Leu^299∗^ residues in addition to those of Trp^31∗, 89∗, 121∗^ and Gln^58∗^ (Fig. 5*C*). Relative orientations of the two ethylene glycol molecules, which approximate aldose fragments, support the interpretation that Leu^294∗^ prevents longer C3-aldoses from adopting a conformation favorable for catalysis (Fig. 7*D*). The ligand butanol, which can mimic the backbone of a C4 aldose with a hydroxyl replacing the aldehyde group, binds into the barley AR in a way that places its OH group about 2 Å too far from the position expected for the aldehyde group. This unfavorable configuration is likely due to the restraint imposed by Leu^295^ (equivalent to ZmAKR4C13 Leu^294∗^) in that enzyme (Fig. 7*E*). The position 294^∗^ plays a key role in aldose binding as indicated by similar results for a rice AKR (22). Also (48); observed 30- and ∼70-fold increases in k_m_'s for glyceraldehyde and xylose, respectively, when HsAKR1B1 Cys^298^ (equivalent to Leu^294^) was mutated to Ala. Collectively, these data support the hypothesis that Leu^294∗^ contributes to the specificity of ZmAKR4C13 for small aldehydes.

## Discussion

The first two portions of this research delineated a 15-member AKR4 gene family in maize (Fig. 1, Table 1) and began bridging the sequence-to-function gap for some of its enzymes (Figure 2, Figure 3, Figure 4). The three phylogenetic subgroups of maize AKR4s were compatible with recent sequence-based efforts by others to define relationships within plant AKR families of Arabidopsis and Medicago (18), tomato (17), diverse plant species (16), and the plant kingdom overall (19). Key challenges have included the incompleteness of valuable resources such as genome sequences, inaccuracies arising from autoannotations, and confounded relationships from autogenerated phylogenetic trees. Prior to the present analyses, these difficulties had obscured the presence of an AKR4C Zm-1 subgroup in maize. Another complication has been the difference between entire classes of AKRs in monocots and dicots (19). A broader functional context can thus be achieved by including biochemically validated genes for AKRs from dicot species in graminaceous phylogenies like that of maize (Fig. 1, Table 1). A similar approach was also used for wheat in recent work by Krishnamurthy *et al.* (Supplemental Data in 2022).

The AKR4C Zm-1 subgroup attracted our particular interest. In part, we were curious about this previously obscured set of maize AKRs. More importantly, though, this clade included the ZmAKR4C13 with its potential for embryo-specific roles including detoxification and possible signaling impacts that rose with its expression at both the mRNA and protein levels during seed maturation (Figure 2, Figure 3, Figure 4). The strong embryo specificity of ZmAKR4C13 and its rise with onset of desiccation extended across NAM-parent lines and indicated a genetic variability of potential use (Fig. 4). This embryo specificity and association with dormancy was also observed for orthologs like the HvAKR4C1 in barley and the ZvAKR4C3 in oats (Table 1 and (3, 5, 54). A further relationship with seed viability was implicated for the AKR4C3 in oats (54) and supported in rice, where activity of an embryo AKR with a similar expression profile (55) correlated with a phase change in viability of stored seeds and carbamylation of proteins (69). A more specific contribution to desiccation tolerance was suggested by the ABA-inducibility of the group-1 AKR4Cs in barley (3), oats (5), and in bromegrass cell cultures (4). Although these orthologs of the AKR4C Zm-1 subgroup are essentially embryo-specific, one from an African resurrection plant (*Xerophyta viscosa*) can be induced under severe desiccation stress of mature leaves (6). Still, the underlying biochemical mechanisms remain elusive. Initially, the possible involvement of ZmAKR4C13 in embryo metabolism of sorbitol produced in kernels was considered, particularly given the demonstrated capacity for AKRs to mediate such a conversion in humans (15). Nonetheless, work here indicates that instead of metabolizing sorbitol, ZmAKR4C13 prefers small aldehyde substrates such as MG. The capacity to utilize these substrates suggests that ZmAKR4C13 may contribute to the detoxification of stress-induced metabolites in developing or desiccating maize seeds.

Potentially protective responses to diverse stresses are also emerging for the second clade of maize AKR4s (AKR4C Zm-2 subgroup) and orthologs in other species. Stresses can produce toxic metabolites from metabolic imbalance, reactive oxygen formation, and a combination of both. Resulting damage from reactive aldehydes can be prevented by their detoxification, so enzymes of the AKR4C Zm-2 subgroup could have invaluable roles in reducing impacts of oxidative stress. Not only are the maize AKR4C’s in this group expressed at sites and times consistent with such a suggestion (Figs. 2 and 3) but analysis of the maize AKR4C7 shows that it can target a broad range of substrates (9). Expression profiles of Arabidopsis orthologs of the AKR4C Zm-2 subgroup closely resemble those in maize, with some members exhibiting constitutive expression while others play roles that are more specialized. Notably, AKR4C8 and AKR4C9 are strongly upregulated in response to osmotic, saline, oxidative, and pathogen-induced stress in Arabidopsis. Similar to maize AKR4C7, these enzymes metabolize a broad spectrum of aldehyde substrates (1), extending to steroid substrates (1) and components of the nitric oxide signaling system, such as S-nitrosoglutathione and S-nitroso-coenzyme A (70). Comparable substrate versatility has been observed in other species, such as Arabidopsis, *D. purpurea*, *Lycopersecum esculentum, Echinochloa colona*, among others (8, 30, 36, 38, 51, 59). These findings highlight the functional diversity of AKR4C Zm-2 subgroup enzymes across different plant species and their adaptive roles in stress response and metabolism.

The third clade in maize was an AKR4A/B Zm-3 subgroup (Figure 1, Figure 2, Figure 3, Figure 4, Table 1) typified by its diversity and abundance of members related to *Dmas1,* a gene required for biosynthesis of chelators used by grasses for uptake and transport of iron (34, 35). Not surprisingly, this AKR4 subclade has expanded in the grasses and grains (19), where it now includes several DMAS-like sequences. Although there is variation among graminaceous DMAS enzymes, the substrate-binding domain is strictly conserved among the true DMAS proteins. All tested thus far can catalyze conversion of the 3′-keto precursor *in vitro* and are upregulated by Fe deficiency (34, 35). Even without deficiency, these proteins and related family members have proposed roles in iron-handling throughout the plant (35) consistent with the broad pattern of expression for some of these AKR4A/B Zm-3 enzymes in maize (Figs. 2 and 3). Other members in this subgroup could include the especially diverse functions typical of this clade. Several maize genes that have *DMAS*-like sequences may or may not encode true DMAS enzymes. The diversity of functions among orthologs of the AKR4A/B Zm-3 subgroup from other species is highlighted by presence of genes for biosynthesis of codeine, cocaine, ascorbic acid, and flavonoids that include phytoalexins (Table 1 and citations therein).

In-depth focus was next directed to the ZmAKR4C13 due to (i) the previously obscured presence of an AKR4C Zm-1 subgroup in maize; (ii) the high and rising levels of *ZmAkr4C13* expression in embryos during maturation; and (iii) the possibility that this AKR could metabolize a range of substrates from MG and toxic aldehydes to diverse sugars. The combined results for expression of ZmAKR4C13 (Figure 2, Figure 3, Figure 4) and its capacity to metabolize MG (Table 2) have important implications for its embryo-specific activity. First, a backup role for AKRs working alongside glyoxylase systems to detoxify MG (13, 14, 39) may be more significant in seeds, where the levels of this stress metabolite can be several times higher than in the rest of the plant (45, 71). Also, constraints to rising levels of MG can enhance desiccation tolerance in tissues as well as seed longevity, as observed for overexpression of AKR4s in tobacco and rice (42, 61). Onset and release of desiccation tolerance also follow changes in expression of native AKR4s at mRNA and protein levels (5, 6) and in seeds, potential contributions to dormancy are further controlled by ABA and gibberellic acid responsiveness of ZmAKR4C13 orthologs (3). This desiccation tolerance was initially thought to involve the potential for sorbitol production by an embryo AKR4C operating as observed for a human ortholog (3, 15). Later the possibility also arose that AKR activity in the embryo might metabolize the sorbitol formed in endosperm by sorbitol dehydrogenase (52). However, our data ruled out both possibilities, as ZmAKR4C13 is unable to utilize either glucose or sorbitol. Instead, its demonstrated ability to metabolize MG suggests a more plausible role for ZmAKR4C13 in contributing to desiccation tolerance in maize seeds.

The possible role of ZmAKR4C13 in aiding detoxification of MG also has implications for production of aflatoxin by the mold species, *A. flavus, A. parasiticus* and *Fusarim* spp. Although these molds are common, they can be present without producing aflatoxin until exposed to MG from stress metabolism by infected hosts like grains (72, 73). The resulting aflatoxin is one of the strongest-known natural carcinogens (72, 73), so focus has been directed to potentially minimizing its formation by limiting levels of endogenous MG. Possible roles of AKRs were initially overlooked due to the well-known prominence of glyoxalases in MG metabolism and the presence of a glyoxylase among candidate enzymes for aflatoxin resistance in maize (74). Nonetheless, recent identification of AKRs as “a second line of defense” for detoxifying MG (39), together with the *Aspergillus*-induced upregulation of ZmAKR4C13 in maize embryos (Fig. 4), opens the possibility of AKR contributions to MG control.

Still other insights into potential functions have emerged for ZmAKR4C13 given its capacity to metabolize MG. Effects of MG as a signaling molecule are emerging in work on human systems, where demonstrated impacts extend beyond general glycation damage to include modification of specific sequences that initiate signals (11, 40). In plants, recent evidence increasingly points toward roles for AKRs consistent with direct or indirect signaling impacts by MG (30, 39) or nitric oxide, the latter transduced by AKR reduction of s-nitrosoglutathione and/or s-nitroso-coenzyme A (41). In addition to the impacts of MG on seed germination (71) and fruit ripening (43) are AKR-associated effects on fruit development that extend from flavor constituents to fruit size and sugar content (30). Although involvement in nitric oxide homeostasis has thus far been attributed to plant AKRs in a different clade (Arabidopsis orthologs of the AKR4C Zm-2 subgroup) (41), the ZmAKR4C13 may also contribute by similar mechanisms. Key components of nitric oxide signaling (both s-nitrosoglutathione and s-nitroso-coenzyme A) can be reduced by the plant AKRs tested (41), and potential for use of the s-nitrosoglutathione substrate is further supported by capacity for the small, 3-C glycine of glutathione to bind at the AKR active site (75).

The ZmAKR4C13 crystal structure (Figure 5, Figure 6, Figure 7) anchors structure-function relationships for this enzyme and provides a “ground truthing” for predictive programs that target protein function. A reference conformation is presented for ZmAKR4C13 that helps validate and strengthen incorporation of estimated 3-dimensional structures (like those predicted using AlphaFold) into functional predictions for enzymes of the large and particularly difficult AKR superfamily (relevant across all species). An architectural mechanism is revealed here that underlies a constraint in substrate size for AKRs. Analysis of the active site, its modifications, and interaction with alternate substrates, indicates that the spatial bulk of Leu at position 294^∗^ of ZmAKR4C13 is instrumental to preventing aldoses with 4-C or more from serving as effective substrates under physiologically relevant conditions. A similar mechanism was proposed for a rice AKR orthologous to a different subgroup (22) also supporting the importance of a bulky amino acid like leucine in this loop of the active site. This hypothesis could be further investigated in future studies by mutating Leu^294∗^ to alanine and assessing the impact of this mutation on substrate specificity. Collective evidence here demonstrates the strong specificity of ZmARK4C13 for small-aldehyde substrates and an expression at sites, times, and a magnitude facilitating roles in stress tolerance, development, and possible signaling.

## Experimental procedures

### Identification of maize AKRs and orthologs

Using the maize AKR4C7 or ZmAKR4C7 amino acid sequence [GenBank: DQ517521] we screened each improved version of the maize genome database (www.maizesequence.org) using BLASTp. The emerging AKR4C subfamily included 15 genes predicted to encode full-length maize proteins. Instances of ambiguous annotations were typically resolved by updated genome releases (especially version 5) and in the present instance by sequencing a full-length cDNA (AKR4C13). A close analysis and revision of the gene model for ZmAKR4C13 was also needed (see Results).

All 15 sequences were aligned using ClustalX version 1.83 (90) and MUSCLE 3.7 (77). For a functionally anchored phylogeny, resulting maize sequences were combined with those of other species in which putative function of a given AKR4 had been confirmed (Fig. 1, Table 1). Phylogenetic relationships were determined by PhyML v. 2 at LIRMM (http://phylogeny.lirmm.fr) (78) using maximum-likelihood analysis and an approximate likelihood-ratio test for statistical evaluation of branch support values. The phylogenic tree was visualized using iTOL v. 6.5.1 (Itol.embl.de) (79).

### Plant material

Maize (*Z. mays* L.) plants (W22 inbred) were grown under standard greenhouse conditions. All were individually hand pollinated. Kernels were harvested at 10, 15, 20, and 25 DAP, frozen in liquid nitrogen, and stored at −80 °C until use. Kernels were manually dissected into glumes, pedicel with transfer region, embryo, embryo proximal region, and endosperm with pericarp. Vegetative structures (roots, leaves, and coleoptiles) were harvested from 10-day-old seedlings germinated under controlled conditions. For infection experiments, the fifth leaf of 3-week-old Golden Queen sweet corn plants (former Monsanto, current Bayer) was inoculated either with *A. flavus* or mechanically damaged as a control. Maize kernels were then harvested at 12 DAP.

### Expression at mRNA and protein levels

Functional insights were sought through temporal and spatial expression of maize genes encoding AKR4s. Paired datasets for transcript and protein abundance were available from (53). Although both were linked to version five of the B73 maize genome at MaizeGDB.org, the data were most directly accessed at qTeller.maizegdb.org using gene identifiers from version 4 (available at MaizeGDB.org under history). All were updated to version 5, including data for the ZmAKR4C13 and its paralog. Data were cross-checked with peptide sequences downloaded from (53). Results were further explored for selected genes with qPCR as below.

### RNA extraction and PCR

Total RNA was extracted from subsamples of those above using a combination of phenol/chloroform and a TRIzol procedure (Invitrogen). Prior to qPCR, total RNA was treated with DNA-Free Reagent (Ambion) to remove possible genomic DNA contaminants. Gene expression was normalized relative to that of 18S rRNA analyzed in separate reactions using TaqMan Ribosomal RNA Control Reagents-VIC Probe (AB Applied Biosystems). Reactions for the first strand were prepared using TaqMan Reverse Transcription Reagent (AB Applied Biosystems, Roche). A SYBR green PCR Master Mix (AB Applied Biosystems, Roche) was used for the second step, together with primers (10 μM each) listed on Table S1 and cDNA (50 ng).

### Fungal inducibility and genetic variation of ZmAkr4C13 expression

The extent of genetic variation was determined for degree and timing of *ZmAkr4C13* expression in developing kernels of NAM-line parents (24 of 25 available) by extracting and analyzing data from (57) (accessed *via* Maize Genomics Resources at http://maize.uga.edu/gene_expr_analyses_download.shtml).

### Generation, purification, and identification of recombinant ZmAKR4C13 aldose reductase

The complete coding sequence of maize ZmAKR4C13 (GRMZM2G169943 RefGen v3, Zm00001eb290330 v5) was PCR-amplified from an embryo cDNA. The sequence of this template cDNA clone was deposited in NCBI together with the corresponding annotation (GU384676). The primers 5′- GCCATGGCGAGTGCACAGGC -3′ (sense) and 5′- GCTCGAGGTCCTCGTGGTCCCACAC -3′ (antisense) were designed for cloning into pET28a. The recombinant plasmid was expressed in *Escherichia coli* BL21(DE3) pRIL, and the protein, featuring a C-terminal His-tag, was purified using conventional Ni-NTA resin. The cleared supernatant was utilized for protein purification *via* immobilized metal affinity chromatography. Final purification was done using a Q-sepharose FF anion exchange chromatography column (Amersham Biosciences), with an AKTA-FPLC System (Amersham Biosciences), as described by (9). Both flow through proteins and eluted fractions were used to estimate total protein concentration and to analyze purity by SDS-PAGE (Fig. S4). Purified maize AR was quantified based on absorbance at 280 nm, using a calculated extinction coefficient of 1.533 g L^−1^ cm^−1^ (80).

Identity of the recombinant ZmAKR4C13 was analyzed by matrix-assisted laser-desorption ionization time-of-flight mass spectrometry (ABI 4700 Proteomics Analyzer) at the Interdisciplinary Center for Biotechnology Research (ICBR)–University of Florida, USA.

### Enzyme activity assays

Maize ZmAKR4C13 activity was assayed spectrophotometrically in a thermostated Hewlett-Packard 8453 spectrophotometer at 30 °C. Reduction of NADP or oxidation of NADP(H) were quantified at 340 nm using a molar extinction coefficient of 6220 M^−1^ cm^−1^ and substrates at either 0 to 20 mM (for DL-glyceraldehyde, MG, or p-nitrobenzaldehyde), or 10 mM to 1500 mM (for D-xylose, D-ribose, D-arabinose, D-glucose, or D-sorbitol). Reactions were assayed in 50 mM sodium phosphate at pH 7.0. The concentration of NADP (for D-sorbitol), and NADP(H) (for the other substrates) was kept constant at 0.25 mM in all experiments and 10 μg of the recombinant maize AR was used for each reaction (400 μl). Blanks contained no substrate. Each assay was replicated three times.

### Protein crystallization

Initial crystallization was done with 10 mg ml^−1^ of the ZmAKR4C13 protein and the β-NADPH cofactor (1:22 mol:mol) using hanging-drop vapor diffusion at 18 °C with Basic and Extension Screens from Sigma-Aldrich. Promising conditions were further optimized by varying pH and precipitant concentration. Nonetheless, only small crystals (needles) were recovered. Thus, several other crystallization screens were employed including (i) index and PEG/Ion from Hampton Research, (ii) the PACT Suite from QIAGEN, and (iii) Crystal Strategy Screen I and II from Molecular Dimensions. These were used to search for better crystals by the sitting-drop method using a nanodrop mosquito crystallization robot. The best hits (*P*2_1_ crystals) emerged under A1 and A2 conditions of the PACT Suite composed of 25% (w/v) PEG 1500 and 0.1 M succinic acid–phosphate–glycine buffer pH 4 or 5, respectively. However, crystals still grew as needles even after refinement of conditions. Seeding assays improved crystal size but the most effective approach involved the Additive Screen from Hampton Research. The refined crystallization condition included 15% (w/v) PEG 1500 and 0.1 M succinic acid–phosphate–glycine buffer pH 4.5 added with 0.01 M of β-NADP^+^. This allowed formation of bigger crystals suitable for X-ray diffraction experiments. A different crystalline form (*P*2_1_2_1_2_1_ crystals) was observed under the D5 condition (0.1 M sodium acetate pH 4.5 and 25% m/v PEG3350) described in the kit Index (Hampton Research). No promising crystallization conditions were identified for the apoenzyme.

### X-ray data collection, structure solution, and refinement

Crystals were cryoprotected using the reservoir solution supplemented with 20% (v/v) ethylene glycol and flash cooled in liquid nitrogen. The X-ray data were collected remotely at 100 K using an ADSC Q210 CCD detector on the beamline ID14-1 at the European Synchrotron Radiation Facility. A total of 360 images at 0.5° rotation per image were collected at the X-ray wavelength of 0.9334 Å. Diffraction data were indexed, integrated, and reduced using XDS (*P*2_1_ crystal) or MOSFLM (81) and SCALA (82) (*P*2_1_2_1_2_1_ crystal). Structure of the holoenzyme in the *P*2_1_ space group was solved by the molecular replacement method using the automated procedure implemented in the BALBES software pipeline (83) available in the YSBL web server (http://www.ysbl.york.ac.uk/YSBLPrograms/index.jsp) and the structure of the aldose reductase from barley (AKR4C1, PDB ID: 2BGS) (20) as a probe model. The first ZmAKR4C13 model, with two protein molecules in the asymmetric unit, was built using ARP/wARP (84) subsequently refined by manual rebuilding in COOT (85), followed by automated refinement with REFMAC5 (86). The final model was used as a template in the MOLREP program (87) to solve the crystal structure of the *P*2_1_2_1_2_1_ crystal form by MR. Water molecules were introduced using the COOT Find Waters tool and *F*_*o*_*-F*_*c*_ map, and further checked manually. Noncrystallographic symmetry restraints were applied in the middle stages of refinement for residues 13 to 318 of each protein chain and between the cofactor molecules. According to MolProbity analyses (88), about 99% and 98% of residues from the protein chains modeled in the *P*2_1_ and *P*2_1_2_1_2_1_ crystals, respectively, were in the favored region of a Ramachandran plot. Additional data collection and refinement statistics are shown in Table 2. Coordinates have been deposited in the RCSB PDB (http://www.rcsb.org) (89) under the accession codes 5JGY and 5JGW for *P*2_1_ and *P*2_1_2_1_2_1_ crystals, respectively.

### Structural analyses

Structural comparisons between the ZmAKR4C13 crystallographic models as well as with homologous structures available in the PDB were performed using the PDBeFold program (67) available at the European Bioinformatics Institute web server (http://www.ebi.ac.uk/msd-srv/ssm/). The Protein Interfaces, Surfaces and Assemblies service PISA (65) at European Bioinformatics Institute web server (http://www.ebi.ac.uk/msd-srv/prot_int/pistart.html) was used to evaluate structural characteristics of the ZmAKR4C13 model described in this work. Structural images were generated using the PYMOL program (90) and the active site cavity boundaries were defined according to CASTP analysis (91).

## Data availability

ARK4C13 structures have been deposited to the Protein Data Bank under PDB IDs 5JGW and 5JGY.

## Supporting information

This article contains supporting information.

## Conflict of interest

The authors declare that they have no conflicts of interest with the contents of this article.

## Figures and Tables

**Figure S4:**