# Supplementary material for: Functional genomics and structural insights into maize aldo-keto reductase-4 family: Stress metabolism and substrate specificity in embryos
Source: J Biol Chem. 2025 Jun 20;301(7):110404. doi: 10.1016/j.jbc.2025.110404 (PMC12302338; doi:10.1016/j.jbc.2025.110404)
Supplement: Legend Supplementary Figures [file mmc1.docx]

**SUPPLEMENTARY INFORMATION**

**Fig. S1.** Comparison of amino acid sequences of the maize and human (AKR1B1 - NP_001619.1) AKRs. The multiple sequences were aligned using the CLUSTALW program. Some important active site residues are indicated in yellow.

**Fig. S2.** Structural alignment of AKR4C13 chains modelled in the asymmetric unit of *P*2_1_ and *P*2_1_2_1_2_1_ crystals. Numbers represent the first and last residues of each modelled chain (A-D).

**Fig. S3.** Comparative analysis of the substrate-binding site of AKRs. Surface representation of the substrate-binding site of AKR4C13 highlighting the invariable residues *(green)* according to a sequence alignment with *Hv*ALR1 (*seq), AKR4C8 (*seq), AKR4C9 (*seq), AKR4A2 (*seq), AKR1A2 (*seq), AKR1B1 (*seq). Catalytic residues are marked with a *. The sequence alignment show only the residues composing the substrate-binding site. Numbers represent the amino acid position at AKR4C13. Colored box highlights conserved residues and green circles indicate those conserved in all compared sequences.

**Fig. S4.** SDS-PAGE (15% gel) of the purified maize aldose reductase (AKR4C13). (1) Molecular mass marker (Dual Color Standards, Bio-rad), (2-5) Purified AKR4C13 (1.926 ug/uL) 0.5, 1.0, 2.0, 5.0 and 10 ul, respectively. The gel was stained with Standard Coomassie protocol. AKR4C13 Molecular Weight: 35659.7 Da and Theoretical pI: 6.47.
