# Supplementary material for: Functional genomics and structural insights into maize aldo-keto reductase-4 family: Stress metabolism and substrate specificity in embryos
Source: J Biol Chem. 2025 Jun 20;301(7):110404. doi: 10.1016/j.jbc.2025.110404 (PMC12302338; doi:10.1016/j.jbc.2025.110404)
Supplement: Figure S1 [file mmc2.pdf]

```
AKR4C13_Zm00001eb290330      ---------------MASA------QA-------------VGQGER--GHFVLKSG---- 20
AKR4C13_Zm00001eb429390      ---------------MPATTAARRSFR-------------TGQSPAGAASFLLHLKSLPS 32
DMAS2_Zm00001eb081580        MHILNFSTAYTFYRALATPDKHRQTRERHAMSSPAARSITCYSKIPE--FLVGT------ 52
DMAS7_Zm00001eb423450        ---------------MASPPR------------NVGTRTTSNPEIPE--FLVGP------ 25
AR5_                         --------------------------------MAATRS---TTAVPELALPSGN------ 19
DMAS8_                       -------------------------------MASTGTT---AAAVPEVTLRSGN------ 20
AR4_                         -------------------------------MASAG-T---TAVVPEVALRSGN------ 19
DMAS6_Zm00001eb419890        -------------------------------------MAGVSASIPC---VVLN------ 14
DMAS1_Zm00001eb010040        ---------------MS---------------------------------ATGR------ 6
DMAS5_Zm00001eb025990        ---------------MAAGGAD----Q------RGNNAATTTFVVPA---VALS------ 26
AKR1B1_human                 ----------------------------------------------MASRLLLN------ 8
AKR4C9_Zm00001eb147990       ----------------------------------------------MATYFTLN------ 8
AKR4C10_Zm00001eb289560      ----------------------------------------------MAESFVLN------ 8
AKR4C8_Zm00001eb347660       ----------------------------------------------MAESFVLS------ 8
AKR4C7_Zm00001eb148000       ----------------------------------------------MARHFVLN------ 8
AR2_Zm00001eb289570          ----------------------------------------------MATYFVLN------ 8

AKR4C13_Zm00001eb290330      ---------------HTIPAVGLGTWRAGSDTA---------------------HSVRTA 44
AKR4C13_Zm00001eb429390      PVAAAASLLSAPRHLHDHPFAACVLYRVARARLFPLLPPLLAALPVAVIPPSMLLPVPAN 92
DMAS2_Zm00001eb081580        -------------NGQPMPAVGLGTASHP----FV---------------AEDVRTSVLT 80
DMAS7_Zm00001eb423450        -------------AGRPVPAVGLGTASFP----FV---------------EENVRTAVLA 53
AR5_                         -------------A-RPVPVIGLGTAVMF----QP---------------PETTKDAVLA 46
DMAS8_                       -------------G-KPMPMVGMGTASFP----VV---------------HEAVRDAVLA 47
AR4_                         -------------ARTAMPMVGMGTASFP----LV---------------HEAVKDGVLS 47
DMAS6_Zm00001eb419890        -------------TGHAMPVLGFGTGSS-----ST---------------PEDLPATILH 41
DMAS1_Zm00001eb010040        -------------APCGLPRIGLGTAVQG----PR---------------PDPVRAAVLR 34
DMAS5_Zm00001eb025990        -------------LGKPMPRVGFGTATATLGQAEG---------------RAGVTEAILR 58
AKR1B1_human                 -------------NGAKMPILGLGTWKSP---------------------PGQVTEAVKV 34
AKR4C9_Zm00001eb147990       -------------TGARIPSVGLGTYKAG---------------------PGVVADAITA 34
AKR4C10_Zm00001eb289560      -------------TGARIPSVGLGTWQIE---------------------HGAVSDAIYA 34
AKR4C8_Zm00001eb347660       -------------TGSRIPSVGLGVWQIQ---------------------PDAANDAIYA 34
AKR4C7_Zm00001eb148000       -------------TGAKIPSVGLGTWQSD---------------------PGVVGNAVYA 34
AR2_Zm00001eb289570          -------------TGAKIPSVGLGTWQAD---------------------NGLVGDAVYA 34
                                               *  .

AKR4C13_Zm00001eb290330      IAEAGYRHVDTAAQYGVEKEVGRGLKAAMEGGI--NRKDLFVTSKLWCTELAPDRVRPAL 102
AKR4C13_Zm00001eb429390      SQEDNRRHCAPTS-PSVHAPAPSTSKLLLRGGG------GCWRLRGRCTELAPDRVRPAL 145
DMAS2_Zm00001eb081580        ALELGYRHIDTAGLYASERVVGEAMAEAVSCGLVASRDELFVTSKVWCTQCHPELVLPSL 140
DMAS7_Zm00001eb423450        ALELGYRHLDTASLYRSERAVGDAVAEAARRGIVASREEVFVTTKMWCSQCHPDLVLPSL 113
AR5_                         AIELGYRHFDTASLYGTEKPLGEGVAEAVRRGLIESREEVFVTSKLWCTQCHPDLVVPSL 106
DMAS8_                       AIEVGFRHFDTAFLYGTEKPLGDAVAEALRRGLLRSREELFVTSKLWCSQTHADLVLPSL 107
AR4_                         AIEVGFRHFDTASMYGTEKPLGDAVAEALRRGTLRSREDLFVTSKLWCSQNHPDLVLPSL 107
DMAS6_Zm00001eb419890        AVRLGYRHIDTASMYGTEGAVGAAVSDAVAAGAVTSRADLFITSKLVMKDAHPDRVLPAI 101
DMAS1_Zm00001eb010040        AIQLGYRHFDTAAHYATEAPIGEAAAEAVRTGLVASREDLFVTSKVWCADAHRDRVLPAL 94
DMAS5_Zm00001eb025990        ALDAGYRHFDTAAVYNTEASLGDAVVEAVRAGTVASRDDLYVTSKLWITDAHPGRVLPAL 118
AKR1B1_human                 AIDVGYRHIDCAHVYQNENEVGVAIQEKLREQVV-KREELFIVSKLWCTYHEKGLVKGAC 93
AKR4C9_Zm00001eb147990       AVKAGYRHIDCAPLYKNEKEIGVALNKLFDDGVV-KREDLFITSKIWCSDLAPEDVPLAI 93
AKR4C10_Zm00001eb289560      AVKAGYRHIDSAVAYRNQKEVGLALQKLFEDGVV-KREDLFVTSKLWPGNHAPEDVQEDL 93
AKR4C8_Zm00001eb347660       AVKAGYRHIDCAAAYNNEEEVGLALKKLFEDGVV-KRDDLFITSKLWAANHAPEDVEEGI 93
AKR4C7_Zm00001eb148000       AVKAGYRHIDCARVYGNEKEIGLALKKLFEEGVV-KREDLFITSKLWNDHHAPEDVPEAL 93
AR2_Zm00001eb289570          AVKAGYRHIDCAQAYNNEKEVGFGLRRVLDEGIV-KREDLFITSKLWDINHAAEDVPVAL 93
                                 . **   :     .                          :          *

AKR4C13_Zm00001eb290330      EKTLKDLQLDYLDLYL----IHWPFRLKDGA---------HMPPEAGEVLE--FDMEGVW 147
AKR4C13_Zm00001eb429390      QKTLKDLQLDYLDLYLERTQIHWPFRLKDGA---------HMPPEAGEVLE--FDMEEVW 194
DMAS2_Zm00001eb081580        KESLKNLQMEYVDLYL----IHWPMAVKP-------SKP-HFPMKREDIVP--MDLSGVW 186
DMAS7_Zm00001eb423450        KESLQNLQMDYVDLYL----VHWPIAAKP-------GKP-QFPFKREDIMP--MDLIGVW 159
AR5_                         RQTLENLQMDYLDLYL----IHMPVCQKP-------GPP-VFPARREDARP--FDFKGVW 152
DMAS8_                       RETLENLQMEYVDLYL----IHWPVCLRPA-----GGPP-KFPNRKEDAVP--LDVAGVW 155
AR4_                         RETLKNLQMEYVDLYL----IHWPVCLKP-------GPP-ELPTRKENAVP--LDLAGVW 153
DMAS6_Zm00001eb419890        RESLARLGLDYLDLFL----VHWPVAADEN----------------NKPVP--FDMEGVW 139
DMAS1_Zm00001eb010040        RRTLSNLQMEYVDLYM----VHWPVTMKAGRF--------TAPFTPEDFEP--FDMRAVW 140
DMAS5_Zm00001eb025990        HRTLQNLQMSYVDLFL----IHHPVSMRAPADDEAEGAG-PAVVVKKDLVA--MDMEGVW 171
AKR1B1_human                 QKTLSDLKLDYLDLYL----IHWPTGFKPG-------KEFFPLDESGNVVPSDTNILDTW 142
AKR4C9_Zm00001eb147990       DITLNDLQLDYLDLYL----IHWPFQIKRG-------SE-LSP---ENFVH--LDMPKTW 136
AKR4C10_Zm00001eb289560      CSALEDLRLDYVDLYL----IHGPIRIQKG-------TM-FIP---ENLIP--TDIPATW 136
AKR4C8_Zm00001eb347660       DTTLQDLQLDYLDLYL----IHGPIRIKKG-------TSTMTP---ENFLP--TDIPATW 137
AKR4C7_Zm00001eb148000       NDSLNDLQLEYLDLYL----IHWPFRVKKG-------TN-TSP---ENFVT--PDFPATW 136
AR2_Zm00001eb289570          NGTLKDLQTDYVDLYL----MHWPVRMKKG-------AG-FGP---HAVVP--SDIPATW 136
                               :*  *  .*:**::    :* *                              :.  .*
```

```
AKR4C13_Zm00001eb290330      REMEGLVKDGLVKDIGVCNYTVAKLNRLMRS--ANVPPAVCQMEMHPGWKNDRIFEACKK 205
AKR4C13_Zm00001eb429390      REMEGLVKDGLVKDIGVCFTTVTKLNRLMRS--ANVPPAVCQMEMHPGWKNDRIFEACKK 252
DMAS2_Zm00001eb081580        QAMEECHRLGLAKMIGVSNFTTKKLQELLAI--AKISPFVNQVELNPTWQQKKLIEFCND 244
DMAS7_Zm00001eb423450        RAMEECHRLGLARMIGVSNFTTKKLQELLAI--AKIPPSVNQVEMNPIWQQKRLAEFCKD 217
AR5_                         QAMEECQRLGLARAIGVSNFRTKHLDKMMPF--ATITPAVNQVEVNPVCQQLKLRGYCAE 210
DMAS8_                       RAMEECQRLGLARAIGVSNFTTRHLDKVLAI--AAVPPAVNQVELNPAWQQRTLRAYCAD 213
AR4_                         RAMEECQRLGLAKAIGVSNFTTRHLDGVLAV--ATVPPAVNQVELNPAWQQRTLRAYCAD 211
DMAS6_Zm00001eb419890        HAMEECHRIGLARSVGVSNFSAAKMSRLLAF--AAVPPAVNQVEVNVGWRQEKVREACAK 197
DMAS1_Zm00001eb010040        EAMEECHRLGLAKAIGVCNFSCKKLETLLSF--ATIPPVVNQVEINPVWQQRKLREFCRA 198
DMAS5_Zm00001eb025990        EEMEECHRRGLARAIGVSNFSCKKLEHLLSV--AKIPPAVNQVEVNPYCRQEKVRNFCRA 229
AKR1B1_human                 AAMEELVDEGLVKAIGISNFNHLQVEMILNKPGLKYKPAVNQIECHPYLTQEKLIQYCQS 202
AKR4C9_Zm00001eb147990       QAMERLYGSGKARAVGVSNFSTRKLADLLAV--ARVTPAVDQVECHPGWQQARLRAFCCS 194
AKR4C10_Zm00001eb289560      GAMEKLYHAGKARAIGVSNFSCKKLQDLLAV--ARVPPAVNQVECHPVWQQDKLRKLCQS 194
AKR4C8_Zm00001eb347660       AAMEKLYDSGKARAIGVSNFSCKKLHDLLAV--ARVPPAVNQVECHPVWQQDKLRKLCQS 195
AKR4C7_Zm00001eb148000       GAMEKLYDAGKARAIGVSNFSSKKLGDLLAV--ARVPPAVDQVECHPGWQQTKLHSFCQS 194
AR2_Zm00001eb289570          AAMEELYDAGKARAIGVSNFSSKKLADLLAV--ARVRPAVDQVECHPVWRQGRLRAFCAS 194
                               **     * .: :*:.     ::  ::        * * *:* :    :  :   *

AKR4C13_Zm00001eb290330      HGIHVTAYSPLGSSE--------KNLAHDPLVEKVANKLDKTPGQVLLRWALQRGTSVIP 257
AKR4C13_Zm00001eb429390      HGIHVTAYSPLGSSE--------KNLAHDPLVEKVANKMDKTPGQVLVRWALHRGTSVIP 304
DMAS2_Zm00001eb081580        KGIHVTAYSPLGGQRIS-KL---NPVRQSDILEEIGKARGKSVAQISLRWIYEQGASMVA 300
DMAS7_Zm00001eb423450        KGIHLTAYSPLAGQSTS-KV---NPVMQSEVLQEVAKARGKSVAQISLRWIYEQGASVVV 273
AR5_                         KGIHVQAFSPLGGQSWAAER---NAVLESEVLAEIAKARGKTVAQVSLRWVFEQGVSFVV 267
DMAS8_                       RGVHVAAYSPLGGQNWDGRG---NAVLDSEVLAEIARARGKTVAQVALRWIHEQGVTCIV 270
AR4_                         RGIHVAAYSPLGGQNWDGQG---SAVLDSEVLAAIAKARGKTVAQVALRWIHEQGVTCIV 268
DMAS6_Zm00001eb419890        NGVVVTAFSPLGAIGTAWGS---NAVMESGALEDIAARRGKTIAQVALRWLHEQGVCFVA 254
DMAS1_Zm00001eb010040        KGIQLCAYSPLGAKGTHWGS---DSVMDSGVLHEIAKSKGKTVAQVCLRWVYEQGDCLIV 255
DMAS5_Zm00001eb025990        NGIQLCGYSAMGASGTAWAN---NSVMDSPVLKQIAHARGKTVAQVCIRWVYEQGDCVIV 286
AKR1B1_human                 KGIVVTAYSPLGSPDRPWAKPEDPSLLEDPRIKAIAAKHNKTTAQVLIRFPMQRNLVVIP 262
AKR4C9_Zm00001eb147990       SGVHFSAYAPLGRM---------KAVASDPVVASVAESLGKTPAQVALRWGIQQGQSVLP 245
AKR4C10_Zm00001eb289560      TGVHLSAYSPLGSPGSPGYS--GPNVLSNPVVMSVAERLQKTPAQVALRWGIQMGQSVLP 252
AKR4C8_Zm00001eb347660       NGVHLSAFSPLGSPGSPWIN--GPSVLKNPIVVSVADKLQKTPAQVALRWGIQMGHSVLP 253
AKR4C7_Zm00001eb148000       TGVHLTAYSPLGSPGTTWM---NGNVLKEPIIISIAEKLGKTSAQVALRWNIQMGHSVLP 251
AR2_Zm00001eb289570          QGIHLSAYSPLGSPGTATVK--AGAVLEHPAVVSAAETLGKTPAQVALRWGVQMGHSVLP 252
                              *: . .:: :.              :     :   .    *: .*: :*:  . .   :

AKR4C13_Zm00001eb290330      KSTRDERIKENIQVFGWEIPEEDFRALCGIKDEKRVLTGEELFVNKTHGPYKSATEVWDH 317
AKR4C13_Zm00001eb429390      KSTRDERIKENIQVFGWEIPEDDFRALCGIKDELSEGW---------HGSWGLRLKR--- 352
DMAS2_Zm00001eb081580        KSLKRERLKENIEIFDWELSDEDRFKIGQIAQRKL-VTVQ--NLLCPEGI-SSVDILDVD 356
DMAS7_Zm00001eb423450        KSFGRDRLKENVEIFDWELTNEDRRKISQIPQHKR-VTVL--GILSPDGV-SSVDLAELD 329
AR5_                         KTYKKERLKENLEIFDWELTDEDRVKISRIPQKKL-SGFS--FMFKPEGEFTSVDVSEIN 324
DMAS8_                       KSYNRERLKQNLEIFDWELTDDDRLKISHIPQRKV-VEAS--GLFSQEGEFTSVDPAELN 327
AR4_                         KSYSKERLRQNLGIFDWELTDEERLKISQIPQRKV-VQTS--SLFSQEGEFTAVDPAELN 325
DMAS6_Zm00001eb419890        RSFNKERLKQNMELFDWELSVDDKEKIMGIPQRRA-CRAE--FFLSPDGPYKTLEELWDG 311
DMAS1_Zm00001eb010040        KSFDEGRMKENLDIVDWELSEEERQRISKIPQRKI-NQGR--RYVSEHGPYKSFEELWDG 312
DMAS5_Zm00001eb025990        KSFNQSRMRENLHIFDWELTDDDHRKISELPESRG-NY-D--FLIHESGPYKTAQELWDG 342
AKR1B1_human                 KSVTPERIAENFKVFDFELSSQDMTTLLSYNRNW---RVCALLSCTSHKDYPFHEEF--- 316
AKR4C9_Zm00001eb147990       KSANESRLKENIDLFGWSIPDELCAKFSEIEQVPL-QFLQRWIIMRGGELINQVKVLWSD 304
AKR4C10_Zm00001eb289560      KSADRTRIGENFDIFDWSIPYDLMAKFSAIKQVRL-LKVE--FVVHPNSGYNTLEDLWDG 309
AKR4C8_Zm00001eb347660       KSANESRIKENIDIFGWSIPEDLMAKFSEIKQVRL-LTAE--FVVHPQAGYNTLEDFWDG 310
AKR4C7_Zm00001eb148000       KSTNEERIKQNLDVYDWSIPDDLLAKFSEIKQARL-LRGN--FIVNPESVYKTHEELWDG 308
AR2_Zm00001eb289570          KSTDEGRIRANLDVFGWSLPEDLLAKFSEIEQERL-IRAG--FFVDPDGVFKSIEEFWDG 309
                             ::    *:  *. : .:.:  :    :

AKR4C13_Zm00001eb290330      ED--------------------  319
AKR4C13_Zm00001eb429390      ----------------------  352
DMAS2_Zm00001eb081580        VIEI------------------  360
DMAS7_Zm00001eb423450        IVEM------------------  333
AR5_                         PIEE------------------  328
DMAS8_                       IVEE------------------  331
AR4_                         ILEE------------------  329
DMAS6_Zm00001eb419890        EI--------------------  313
DMAS1_Zm00001eb010040        EI--------------------  314
DMAS5_Zm00001eb025990        EITAGQSNQTPLVSDD------  358
AKR1B1_human                 ----------------------  316
AKR4C9_Zm00001eb147990       MDEELAT---------------  311
AKR4C10_Zm00001eb289560      EVEYEDERTENQDHSDFLRCFS  331
AKR4C8_Zm00001eb347660       EI--------------------  312
AKR4C7_Zm00001eb148000       EL--------------------  310
AR2_Zm00001eb289570          EI--------------------  311
```
